# Association of Vitiligo With Autoimmune Disorders: A Bidirectional Two‐Sample and Summary‐Based Mendelian Randomization Study

**DOI:** 10.1111/jocd.70211

**Published:** 2025-06-04

**Authors:** Jiangfeng Huang, Ling Jiang, Yibo Hu, Chuhan Fu, Keyi Zhang, Yaqing Wen, Shu Zhou, Jinhua Huang, Jing Chen, Qinghai Zeng

**Affiliations:** ^1^ Department of Dermatology, Third Xiangya Hospital Central South University Changsha China

**Keywords:** autoimmune disorders, Mendelian randomization, rheumatoid arthritis, summary‐based Mendelian randomization, vitiligo

## Abstract

**Background:**

The existence of a definite direct causal relationship between vitiligo and diverse autoimmune disorders remains unknown due to the influence of confounding factors and potential reverse causality.

**Methods:**

Mendelian randomization (MR) is a technique employed to explore causal connections between two phenotypes. In our research, bidirectional two‐sample MR analyses were utilized to evaluate the causal connections between vitiligo and multiple autoimmune diseases (systemic lupus erythematosus, Graves' disease, inflammatory bowel disease, alopecia areata [AA], type 1 diabetes mellitus [T1MD], and rheumatoid arthritis [RA]). Furthermore, we utilized summary‐based Mendelian randomization (SMR) analysis to search for common susceptibility loci between two diseases that reciprocally elevate each other's risk. Finally, colocalization analyses were used to validate the robustness of the selected genes.

**Results:**

There was an indication of potential causation between RA and vitiligo (IVW OR = 1.19; 95% CI = 1.05–1.13; *p* = 0.008). Furthermore, evident causal connections exist between vitiligo and AA (IVW OR = 1.14; 95% CI = 1.04–1.26; *p* = 0.008), T1MD (IVW OR = 1.14; 95% CI = 1.06–1.23; *p* < 0.001), and RA (IVW OR = 1.08; 95% CI = 1.03–1.13; *p* < 0.001). In SMR analyses and colocalization analyses, we identified three shared genes associated with both vitiligo and RA, including: FCRL3, FADS1, and FADS2.

**Conclusion:**

Our findings demonstrated that vitiligo and RA mutually act as risk factors for each other. Additionally, vitiligo had significant causal relationships with AA and type 1 diabetes.

## 1 Introduction

Vitiligo, a chronic skin depigmentation condition, is marked by selective melanocyte destruction and affects about 1% of the global population [1]. It manifests as localized or generalized loss of pigmentation in the skin and mucous membranes [2]. Although most patients with vitiligo do not experience evident subjective symptoms, the visible depigmented patches can significantly impact social interactions, work life, daily life, and mental health [2]. The etiology of vitiligo is multifactorial, caused by an interplay of genetics, oxidative stress, inflammation, and various environmental factors [3]. Previous observational studies revealed that there exist causal connections between vitiligo and various autoimmune diseases [4]. Nevertheless, the causal connections between vitiligo and diverse autoimmune diseases remain uncertain because of biases arising from potential confounders and reverse causality in observational studies. Given that vitiligo is an autoimmune disorder marked by immune dysregulation, investigating its causal relationships with various autoimmune diseases is of paramount importance. This research not only deepens our understanding of vitiligo's pathogenesis but also contributes to the advancement of novel therapeutic approaches. Consequently, we necessitate a more robust analytical method to evaluate the causal relationships among them.

Mendelian randomization (MR) analysis is a technique employed to evaluate the causal connections between two phenotypes [59]. It utilizes single nucleotide polymorphism (SNP) found via genome‐wide association studies (GWAS) as instrumental variables (IVs) [5]. MR analysis has the capability to effectively eliminate biases resulting from confounders and reverse causality, given that genotypes are random assignments at conception [59]. Therefore, we used bidirectional two‐sample MR analyses to assess the causal link between vitiligo and multiple autoimmune disorders, such as systemic lupus erythematosus (SLE), Graves' disease, inflammatory bowel disease (IBD), alopecia areata (AA), type 1 diabetes mellitus (T1MD), and rheumatoid arthritis (RA).

Summary data‐based Mendelian randomization (SMR) analysis is an approach conducted to detect genes that were causally connected to diseases using expression quantitative trait loci (eQTL) data and GWAS summary statistics data [7]. The results of SMR analyses for two phenotypes can be integrated to identify common genetic loci.

Colocalization analysis is a statistical approach used to evaluate whether two traits share the same causal genetic variant within a specific genomic region. It helps identify potential causal genes or regulatory elements by assessing the overlap of genetic signals between the traits [8].

## 2 Material and Method

### 2.1 Study Design

To deduce the causal relationships between vitiligo and various autoimmune disorders, we conducted bidirectional two‐sample MR analyses utilizing GWAS data. The analyses were conducted in strict accordance with the STROBE‐MR checklist, which was also completed as part of the study (Table S1). Initially, various autoimmune disorders were considered as exposures in the MR analyses to ascertain their causal effects on vitiligo. Subsequently, we carried out reverse MR analyses to validate the causal connections between vitiligo and autoimmune disorders. The IVs used in MR analyses must comply with the following three principles: (1) The IVs must be closely related to the exposure, ensuring their effective representation of it; (2) the connection between IVs and the exposure should remain unaffected by confounding factors, ensuring the validity of causal inference, (3) the impact of IVs should be realized solely through the effect of the exposure on the outcome, excluding effects from other pathways [9]. Furthermore, the SMR study, employing the top‐SNP in expression quantitative trait locus (eQTL) as the proxy and the GWAS data of diseases as the outcomes, was employed to identify shared risk genes between the two diseases, both of which act as reciprocal risk factors for each other. Finally, colocalization analysis was performed to validate the robustness of the identified genes.

### 2.2 Data Sources

Table 1 presents the GWAS data for diverse diseases gathered from public databases utilized in bidirectional two‐sample MR analyses. Notably, the IVs for vitiligo were extracted from the largest meta‐analysis of GWAS [10]. Except for the IVs for AA, which were sourced from FinnGen (https://www.finngen.fi/en), the IVs for SLE, Graves' disease, IBD, T1MD, and RA were all retrieved from prior GWAS studies [11, 12, 13, 14, 15, 16]. The eQTL summary data used in SMR analyses were sourced from three databases, including the V8 release of genotype‐tissue expression (GTEx) eQTL summary data, eQTLGen, Westra eQTL data, and CAGE eQTL data [17, 18, 19, 20].

**TABLE 1:** The specifics of the GWAS data utilized in MR analyses.

Trait	nSNP	Dataset	Sample	Cases	Control	Year	PMID
SLE	7 071 163	GCST003156	14 267	5201	9066	2015	26502338
Graves' disease	11 831 932	GCST90043624	456 348	210	456 138	2021	34737426
IBD	157 115	GCST003045	27 432	6968	20 464	2015	26192919
Alopecia areata	20 169 959	FinnGen_R9	361 822	682	361 140	2023	
Type I diabetes	12 794 567	GCST010681	24 840	9266	15 574	2020	32005708
RA	13 297 690	GCST90132223	97 173	22 350	74 823	2022	36333501
Vitiligo	9 068 689	GCST004785	40 258	2853	37 405	2016	27723757

Abbreviations: IBD, inflammatory bowel disease; nSNP, number of single nucleotide polymorphisms; RA, rheumatoid arthritis; SLE, systemic lupus erythematosus.

### 2.3 Selection of Genetic Variants

To verify a resilient link between the genetic IVs and the exposure, *p* value criterion of < 5 × 10^−8^ was set for the selection of leading SNPs. However, as none of the SNPs representing Graves' disease and AA meet the stringent criteria (*p* < 5 × 10^−8^), we have chosen a statistical significance cutoff of *p* < 1 × 10^−5^ for them. Then, linkage disequilibrium (LD) clumping (*r* ^2^ < 0.001 within 10‐MB window) utilizing the reference panel was conducted to identify that SNPs were independent [21]. Subsequently, we harmonized the IVs to assure the uniformity of their association effects with the identical alleles in both the exposure and outcome. To eliminate confounding factors, Phenoscanner V2 was used to exclude SNPs that exhibited a significant association with the outcomes (*p* < 5 × 10^−8^) [22]. Finally, the F‐statistics for each SNP, computed as beta^2^/se^2^, were illustrated in Table S2 [21]. Those with an F‐statistic more than 10 were considered significantly associated with the exposure and utilized in subsequent MR analyses [23].

### 2.4 Mendelian Randomization Analyses

To verify the causal connections between autoimmune diseases and vitiligo, we employed four MR approaches: Inverse‐variance weighted (IVW), weighted median, MR‐Egger, and simple median [24, 25, 26]. IVW served as the main approach due to its superior statistical power [25]. When substantial heterogeneity was observed among SNPs, we used the random‐effects model of IVW [27]. Weighted median and simple median can still provide robust estimation results when almost 50% of the weight originates from ineffective instruments [24]. MR‐Egger enables robust estimation assuming that IVs do not directly influence the outcome [26].

### 2.5 Sensitivity Analyses

To ensure the reliability of the results derived from MR analyses, we conduct a variety of sensitivity analyses. Initially, we applied Cochran's Q examination to evaluate the presence of heterogeneity and subsequently determined the appropriate IVW model for bias reduction attributed to heterogeneity [27]. Subsequently, the MR‐Egger intercept test was conducted to identify the existence of directional pleiotropy. Furthermore, in order to comprehensively evaluate horizontal pleiotropy, we performed the MR‐PRESSO to identify potential outliers [28]. Upon detecting significant outliers, we recalculated the causal estimate after their removal. Lastly, leave‐one‐out analysis was used to detect whether a single outlier variant affected the causal effect estimates [29].

### 2.6 Summary‐Based Mendelian Randomization Analyses

SMR analysis is a method used to identify the shared pathogenic genes and the top‐SNPs of those genes. It achieves this by incorporating summary statistics from eQTL studies and GWAS within the MR framework. Initially, we conducted SMR analysis using GWAS summary data and the V8 release of GTEx eQTL summary data [17]. Furthermore, we validated the results by analyzing cis‐eQTL summary data from eQTLGen, Westra, and CAGE in conjunction with GWAS [18, 19, 20].

By default, we chose cis‐eQTLs with a statistical significance cutoff of *p* ≤ 5 × 10^−8^, showing the most robust associations with gene expression, as IVs for SMR analysis [30]. SNPs with allele frequency differences > 0.20 were excluded. Additionally, the heterogeneity in dependent instruments (HEIDI) test was employed to mitigate the impact of pleiotropy [7]. For the test, SNPs with LD *r* ^2^ between top‐SNP > 0.90 or < 0.05 were excluded. Finally, false discovery rate (FDR) *q* value criterion of < 0.05 and HEIDI test *p* value criterion of > 0.05 were used to identify the significant probes [31].

### 2.7 Colocalization Analyses

To assess whether the identified eQTLs are associated with shared genetic loci in both vitiligo and RA, we extracted cis‐eQTL data for FADS1, FADS2, and FCRL3 from eQTLGen and used these data for colocalization analyses with the GWAS results for both vitiligo and RA, focusing on regions extending 300‐kb upstream of the transcription start site and 300‐kb downstream of the transcription end site. The colocalization analysis evaluates five possible scenarios:

H0: SNPs are not associated with either trait.

H1: SNPs are associated with only one of the traits.

H2: SNPs are associated with only the alternative trait.

H3: SNPs are linked to both traits but do not share the same genetic variants.

H4: SNPs are associated with both traits and share identical genetic variants.

A threshold of PPH4 ≥ 0.8 was set for screening.

### 2.8 Software and Packages

The MR analyses were conducted by statistical software R (version 4.3.0) with R packages, including TwoSampleMR (version 0.5.7), MRPRESSO (version 1.0), and coloc (version 5.2.3). SMR analyses were done using SMR (version 1.3.1).

### 2.9 Ethnic

The studies included in this analysis were exempt from requiring ethics approval and informed consent, as they utilized publicly available data. Furthermore, the original studies contributing to this dataset had previously fulfilled these ethical requirements.

## 3 Result

### 3.1 Mendelian Randomization

#### 3.1.1 Selection of Genetic Variants

Table S2 provides an overview of the IVs utilized in the bidirectional MR analyses. Notably, the results of the F‐statistic were all more than 10, indicating that all the SNPs were strong instruments with a low risk of weak instrument bias. Furthermore, Phenoscanner V2 was employed to identify and exclude the SNPs related to outcomes prior to the MR analyses. Among the IVs representing vitiligo, two SNPs (rs7007905, rs772921) were found to be significantly connected to AA (*p* < 5 × 10^−8^), while one SNP (rs2247315) exhibited a significant relationship with RA.

#### 3.1.2 Effect of Autoimmune Disorders on Vitiligo

**FIGURE 1:**
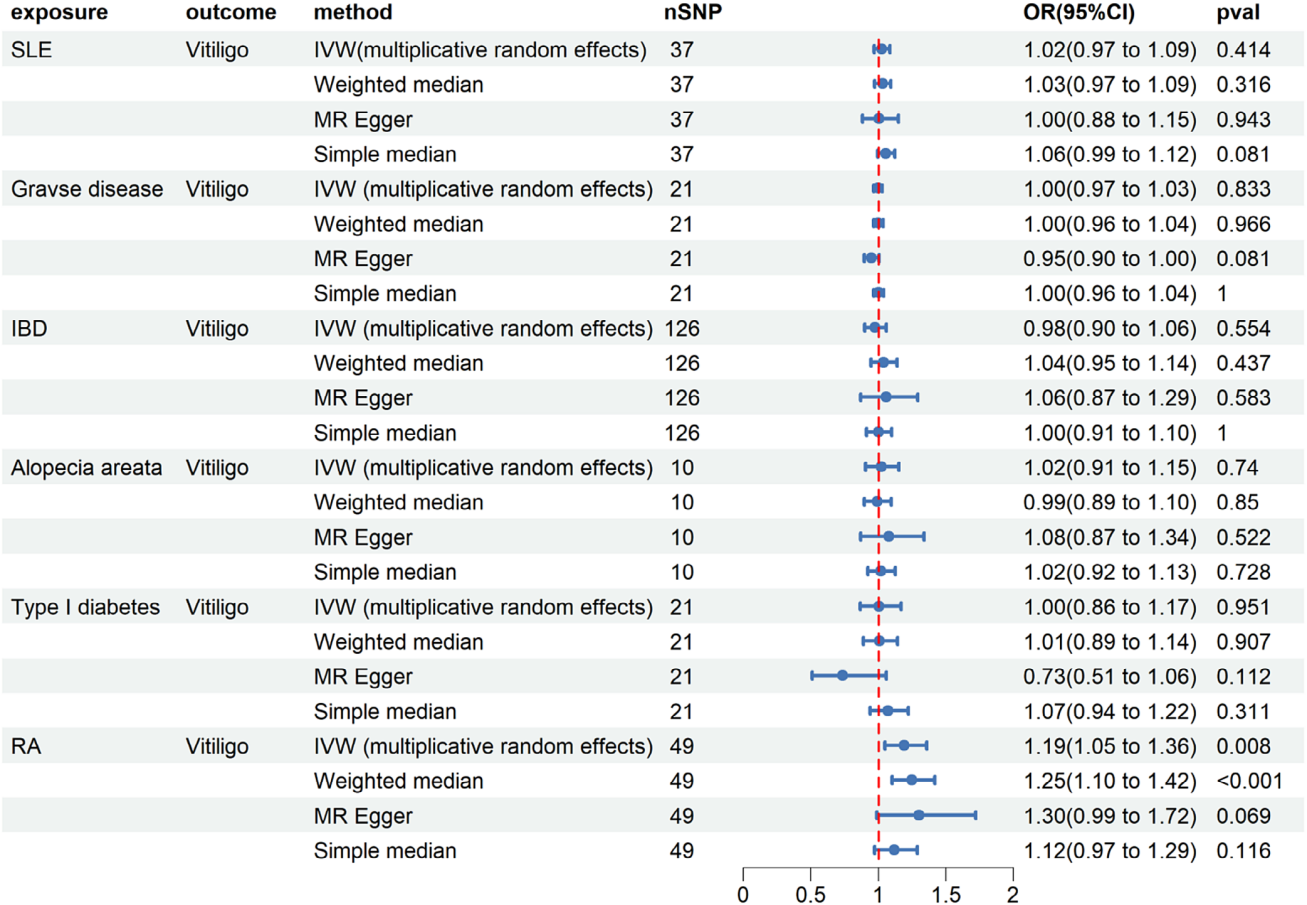
The effect of autoimmune disorders on vitiligo. CI, confidence interval; IBD, inflammatory bowel disease; IVW, inverse‐variance weighted; OR, odds ratio; RA, rheumatoid arthritis; SLE, systemic lupus erythematosus.

The main findings of the MR analyses between autoimmune disorders and vitiligo are illustrated in Figure 1. The results of analyses indicated that the majority of exposures are not statistically significant in relation to the occurrence of vitiligo, yet there existed indicative evidence of a potential causal link between RA and vitiligo (IVW OR = 1.19; 95% CI = 1.05–1.13; *p* = 0.008). Moreover, when we used the weighted median approach, the results were aligned with IVW (OR = 1.25; 95% CI = 1.10–1.42; *p* < 0.001). The scatter plots illustrate the effects of various autoimmune diseases on vitiligo when employing different MR methods (Figure S1).

#### 3.1.3 Effect of Vitiligo on Autoimmune Disorders

**FIGURE 2:**
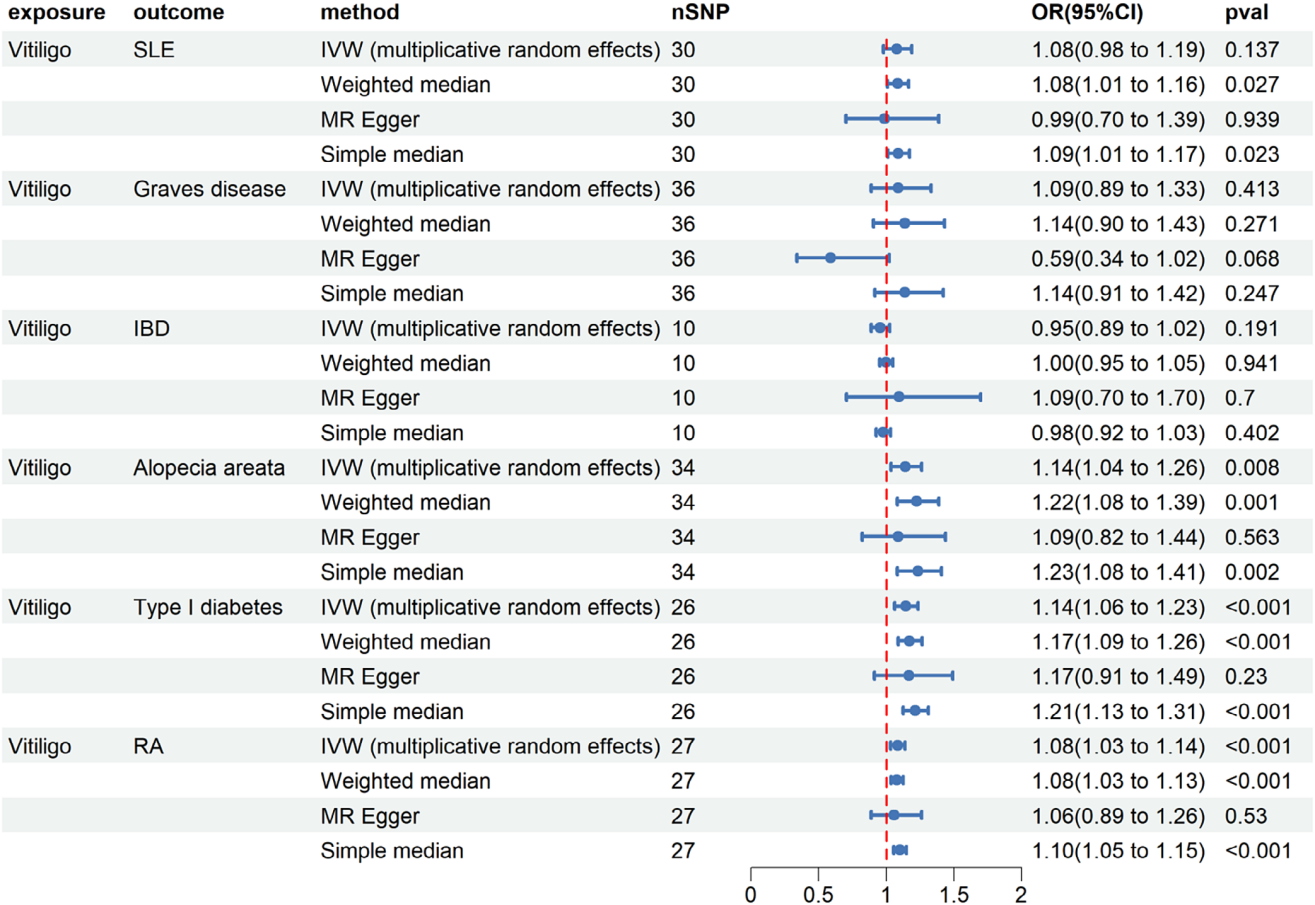
The effect of vitiligo on autoimmune disorders. CI, confidence interval; IBD, inflammatory bowel disease; IVW, inverse‐variance weighted; OR, odds ratio; RA, rheumatoid arthritis; SLE, systemic lupus erythematosus.

As depicted in Figure 2, the results of reverse MR analyses demonstrate that causal relationships between vitiligo and AA (IVW OR = 1.14; 95% CI = 1.04–1.26; *p* = 0.008), T1MD (OR = 1.14; 95% CI = 1.06–1.23; *p* < 0.001), and RA (IVW OR = 1.08; 95% CI = 1.03–1.13; *p* < 0.001) were statistically significant. Similarly, when employing the weighted median method and the simple median method to conduct MR analyses, the causal effects of vitiligo on AA, T1MD, and RA were in line with previous results (Figure 2). The effects of vitiligo on those autoimmune diseases were visualized by the scatter plots (Figure S2).

### 3.2 Sensitivity Analysis

We conducted several sensitivity analyses to validate the reliability of the MR findings. As shown in Table S3, significant heterogeneity was shown in all results (*Q* value < 0.05), we selected the random‐effect IVW approach to address the heterogeneity. When performing the MR‐Egger intercept test, there was no horizontal pleiotropy (*p* < 0.05) in the majority of results except for the causal effect of vitiligo on Graves' disease (*p* = 0.02; Table S4). Most of the results of MR PRESSO analyses present that there exists significant horizontal pleiotropy; yet, outlier‐corrected analysis showed that the ORs were of no noticeable change (Table S5). The leave‐one‐out analyses revealed that there was no individual outlier variant influencing the effect estimates of vitiligo on AA, T1MD, RA, as well as RA on vitiligo (Figure S3).

### 3.3 Summary‐Based Mendelian Randomization Analyses

We performed SMR analyses to identify and explore the shared pathogenic genes associated with both RA and vitiligo. The SMR analysis was employed to infer putatively causal genes of vitiligo by analyzing GWAS summary data and the V8 release of GTEx eQTL summary data. Additionally, the results of the SMR analysis for RA were obtained directly from the previous research [30]. Six shared risk genes were identified, namely FCRL1, FCRL3, FADS1, FADS2, AP003774.1, and CCDC88B. Furthermore, of these, three genes—FCRL3, FADS1, and FADS2—were conclusively identified through the analysis of GWAS summary data and cis‐eQTL data from eQTLGen, Westra, and CAGES (Table S6).

### 3.4 Colocalization Analyses

**FIGURE 3:**
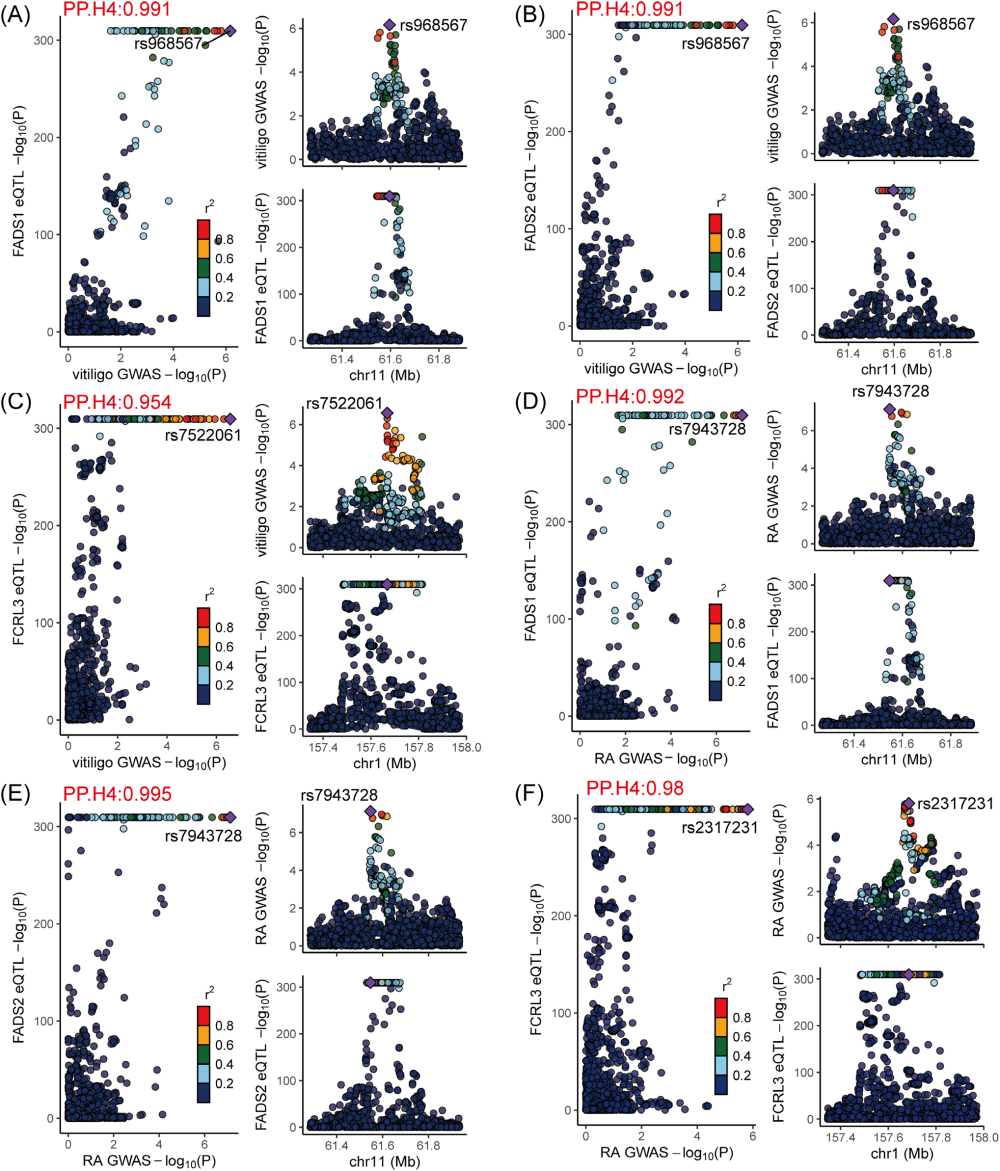
Colocalization analyses of FADS1, FADS2, and FCRL3 with vitiligo and rheumatoid arthritis. (A) The results of colocalization analyses between FADS1 and vitiligo; (B) The results of colocalization analyses between FADS2 and vitiligo; (C) The results of colocalization analyses between FCRL3 and vitiligo; (D) The results of colocalization analyses between FADS1 and rheumatoid arthritis; (E) The results of colocalization analyses between FADS2 and rheumatoid arthritis; (F) The results of colocalization analyses between FCRL3 and rheumatoid arthritis. eQTL, expression quantitative trait loci; RA, rheumatoid arthritis.

The eQTL data of FCRL3, FADS1, and FADS2 were analyzed for colocalization with GWAS data for both vitiligo and RA. The results showed that their PP.H4 were all greater than 0.8, further confirming the robustness of our findings (Figure 3 and Table S7).

## 4 Discussion

In the MR study conducted above, we observed a bidirectional causal relationship between RA and vitiligo. Similarly, vitiligo exhibited an increased risk of AA and T1MD. Furthermore, all outcomes successfully passed sensitivity tests. The SMR analyses and colocalization analyses results suggest that FCRL3, FADS1, and FADS2 are shared genes associated with both vitiligo and RA.

The previous studies have found that individuals with vitiligo may be at a heightened risk of developing RA [32, 33], aligning with our research findings. For example, the most recent systematic review and meta‐analysis of comorbidities in individuals with vitiligo indicated an increased susceptibility to RA among patients with vitiligo (OR = 1.82, 95% CI = 1.55–2.15) [34]. Similarly, previous studies have reported a higher likelihood of AA and T1MD development in individuals with vitiligo than individuals without the condition [34, 35]. However, our analysis results suggest that AA and T1DM are not linked to an elevated risk of developing vitiligo, contrary to some studies suggesting that patients with AA and T1DM are more susceptible to vitiligo [36, 37, 38]. Hence, additional comprehensive research is required to further confirm whether there exist causal relationships.

RA is a systemic inflammatory autoimmune disorder marked by synovitis [39]. One of its key pathogenic mechanisms is the imbalance of CD4+ T cells [40]. Recent research has indicated that the heightened generation of IFN‐γ by CD8+ T cells reveals the significant contributions of CD8+ T cells and IFN‐γ in the onset and progression of synovitis [41]. Furthermore, studies have demonstrated an elevation in CD8+ T cells in the peripheral blood of individuals with vitiligo [42]. There is a notable enrichment of melanocyte‐specific CD8+ T cells in the skin lesions. It is noteworthy that the IFN‐γ‐Chemokine Axis involving CD8+ T cells contributes to the onset and progression of vitiligo [42]. Tissue‐resident memory (TRM) cells, a subtype of memory T cells, reside within tissues for extended periods and play a vital role in peripheral immune surveillance [43]. CD8+ TRM cells have been observed to accumulate within the synovial tissue of inflamed joints in RA patients, persisting even during remission, indicating that TRM cells may contribute to RA relapse [44]. Similarly, TRM cells work as a contributing factor in the recurrence of vitiligo and CXCR3, which is the homing receptor of CD8+ TRM cells, exhibited heightened expression [45]. Additionally, we detected potential shared susceptibility genes between vitiligo and RA through SMR analyses, namely FCRL3, FADS1, and FADS3. Furthermore, prior studies have documented their causal associations with both vitiligo and RA [46, 47, 48, 49]. FCRL3 is a receptor expressed on lymphocytes and has been found to be connected to various autoimmune diseases. FCRL3 stimulation can curb the suppressive function of Treg cells and promote the production of IFN‐γ, leading to immune dysregulation [50]. FADS1 and FADS2 contribute to the regulation of several polyunsaturated fatty acids (PUFAs) [51]. Genetic variations in FADS1 and FADS2 can induce inflammation by modulating the activity of various enzymes and influencing arachidonic acid synthesis [52].

AA manifests as an autoimmune condition leading to non‐scarring hair loss [53]. Autoreactive lymphocytes, including Th1, Th17, NK, and CD8+ T cells, infiltrate around the hair follicles and produce IFN‐γ, which activates the JAK–STAT pathway, disrupting hair follicle function and resulting in hair loss [54]. Cytokines including IL‐2, IL‐15, CXCL10, IL‐12, and IL‐2 are involved during the development of the disease [54]. All of these are remarkably consistent with the pathological mechanisms of melanocyte loss in patients with vitiligo. Additionally, previous research suggested the potential existence of shared pathogenic mechanisms between them [55].

T1DM is marked by the complete absence of insulin because of the autoimmune destruction of pancreatic β cells [56]. The pathogenesis of T1DM involves not only B cell‐mediated production of antibodies against pancreatic β cells but also the activation of β cell‐specific CD8+ T cells [57]. Similar to vitiligo, patients with T1DM also display functional deficits in regulatory T (Treg) cells, which can suppress immune responses [58].

## 5 Conclusion

In general, evident causal associations exist between vitiligo and diverse autoimmune disorders including RA, AA, and T1MD, suggesting that vitiligo might exert a substantial influence on the development of these autoimmune disorders. Additionally, RA is linked to a heightened risk of developing vitiligo. To elucidate the intricate relationships between vitiligo and diverse autoimmune disorders including RA, AA, and T1MD, further research is required to uncover the underlying mechanisms.

## Author Contributions

J.F.H. conceived and designed the study, wrote the original draft, reviewed and edited the manuscript, and was responsible for validation, resources, methodology, investigation, funding acquisition, formal analysis, and data curation. L.J. contributed to funding acquisition, visualization, validation, software development, investigation, and data curation. Y.H., C.F., K.Z., and Y.W. were responsible for validation and resources. S.Z. handled software development and formal analysis. J.H.H. and J.C. oversaw project administration and funding acquisition. Q.Z. reviewed and edited the manuscript and contributed to project administration, funding acquisition, and conceptualization. All authors critically reviewed the manuscript, provided intellectual input, read, and approved the final manuscript.

## Conflicts of Interest

The authors declare no conflicts of interest.

## Supporting information


Data S1.

## Data Availability

The GWAS data employed in this article were derived from studies available online, all of which have undergone ethical review.
