# Supplementary material for: Association of Vitiligo With Autoimmune Disorders: A Bidirectional Two‐Sample and Summary‐Based Mendelian Randomization Study
Source: J Cosmet Dermatol. 2025 Jun 4;24(6):e70211. doi: 10.1111/jocd.70211 (PMC12137767; doi:10.1111/jocd.70211)
Supplement: Supplementary file 1 — Data S1. [file JOCD-24-e70211-s001.docx]

**Contents**

**1. Supplemental Figures**

Figure S1. The scatter plots illustrating the effects of various autoimmune disorders on vitiligo when employing different MR methods

Figure S2. The scatter plots illustrating the effects of vitiligo on various autoimmune disorders when employing different MR methods

Figure S3. The leave-one-out analyses results of vitiligo on alopecia areata, type 1 diabetes mellitus, rheumatoid arthritis and of rheumatoid arthritis on vitiligo

**2. Supplemental Tables**

Table S1. STROBE-MR checklist of recommended items to address in reports of Mendelian randomization studies

Table S2. Overview of the IVs utilized in the bidirectional MR analyses

Table S3. The results of the heterogeneity test

Table S4. The results of MR-egger intercept test

Table S5. The results of MR PRESSO analyses

Table S6. 6 shared risk genes identified in SMR analyses

Table S7. Colocalization analyses

1. **Supplemental Figures**


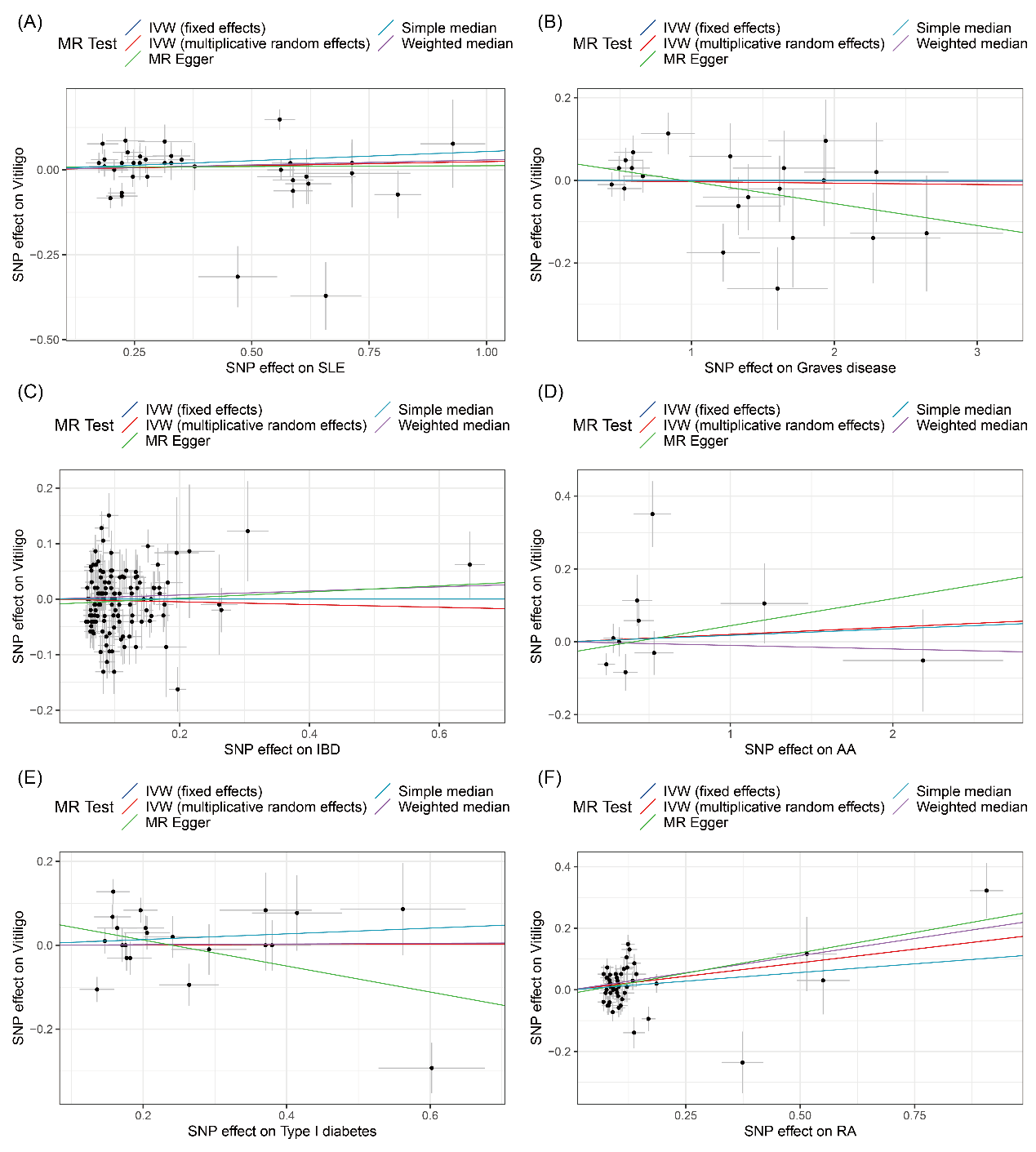


**Figure S1. The scatter plots illustrating the effects of various autoimmune disorders on vitiligo when employing different MR methods.** (A) Scatter plot about the effect of systemic lupus erythematosus on vitiligo; (B) Scatter plot about the effect of Graves' disease on vitiligo; (C) Scatter plot about the effect of inflammatory bowel disease on vitiligo; (D) Scatter plot about the effect of alopecia areata on vitiligo;(E) Scatter plot about the effect of type 1 diabetes mellitus on vitiligo; (F) Scatter plot about the effect of rheumatoid arthritis on vitiligo.

**Abbreviations:** MR, Mendelian randomization; SNP, single-nucleotide Polymorphism SLE: systemic lupus erythematosus; IBD: inflammatory bowel disease; AA: alopecia areata; RA: rheumatoid arthritis.


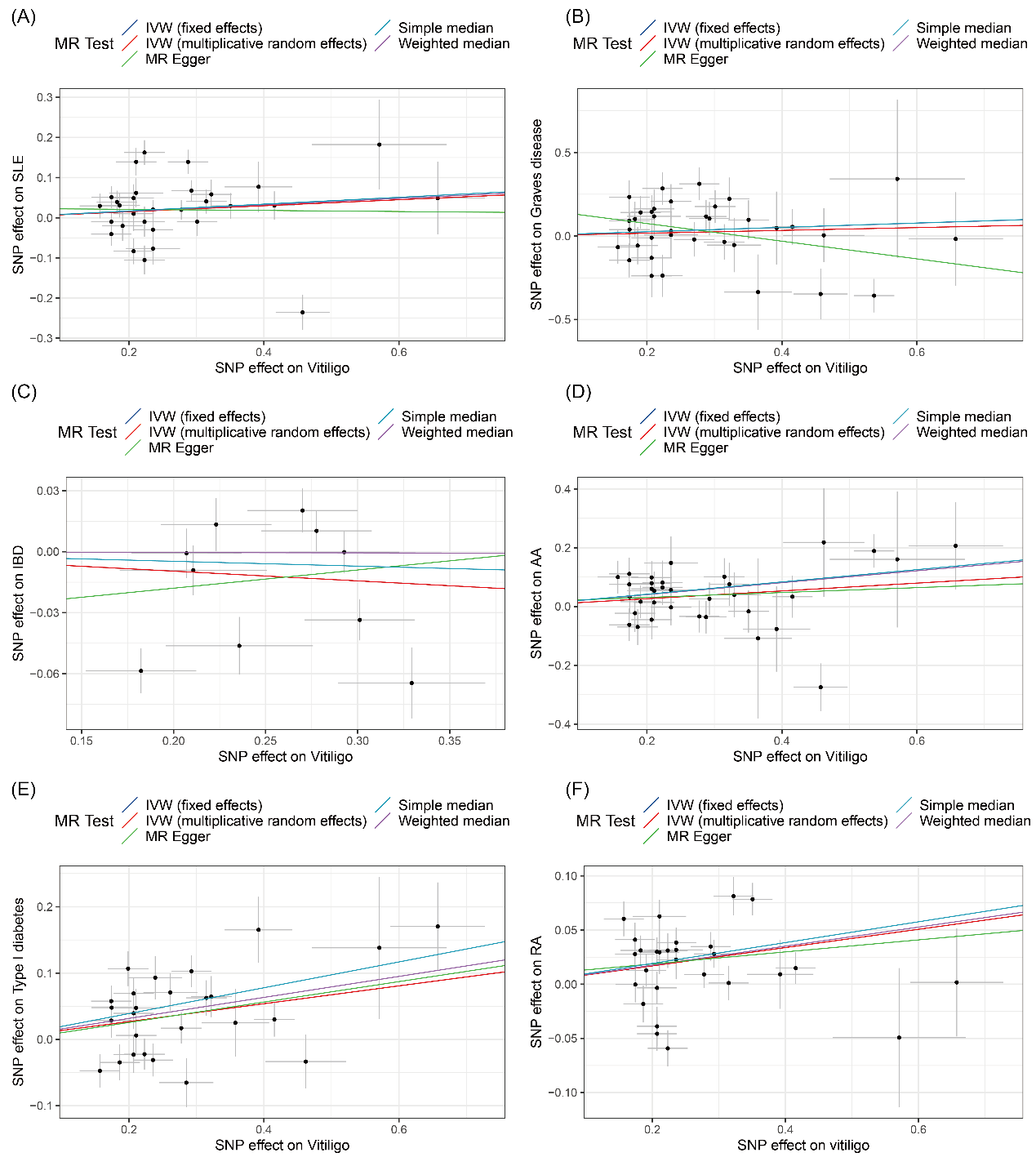


**Figure S2. The scatter plots illustrating the effects of vitiligo on various autoimmune disorders when employing different MR methods.** (A) Scatter plot about the effect of vitiligo on systemic lupus erythematosus; (B) Scatter plot about the effect of vitiligo on Graves' disease; (C) Scatter plot about the effect of vitiligo on inflammatory bowel disease; (D) Scatter plot about the effect of vitiligo on alopecia areata; (E) Scatter plot about the effect of vitiligo on type 1 diabetes mellitus; (F) Scatter plot about the effect of vitiligo on rheumatoid arthritis.

**Abbreviations:** MR, Mendelian randomization; SNP, single-nucleotide Polymorphism SLE: systemic lupus erythematosus; IBD: inflammatory bowel disease; AA: alopecia areata; RA: rheumatoid arthritis.


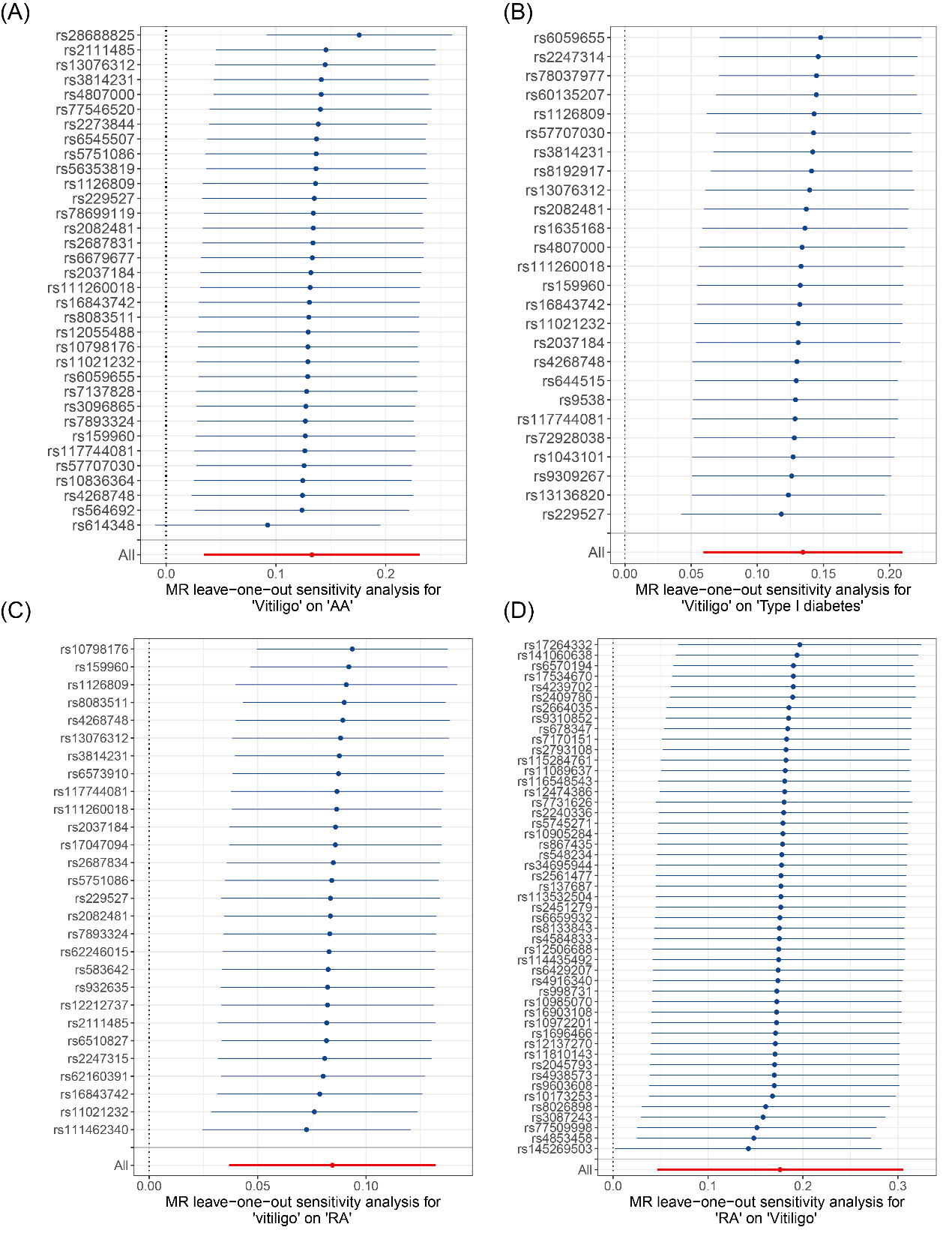


**Figure S3. The leave-one-out analyses results of vitiligo on alopecia areata, type 1 diabetes mellitus, rheumatoid arthritis and of** **rheumatoid arthritis on vitiligo.** (A) leave-one-out plot about the effect of vitiligo on alopecia areata; (B) leave-one-out plot about the effect of vitiligo on type 1 diabetes mellitus; (C) leave-one-out plot about the effect of vitiligo on rheumatoid arthritis; (D) leave-one-out plot about the effect of rheumatoid arthritis on vitiligo.

**Abbreviations:** MR, Mendelian randomization; AA: alopecia areata; RA: rheumatoid arthritis.

1. **Supplemental Tables**

**Table S1. STROBE-MR checklist of recommended items to address in reports of Mendelian randomization studies^1 2^**

| **Item No.** | **Section** | **Checklist item** | **Page No.** | **Relevant text from manuscript** |
| --- | --- | --- | --- | --- |
| 1 | **TITLE and ABSTRACT** | Indicate Mendelian randomization (MR) as the study’s design in the title and/or the abstract if that is a main purpose of the study | 1 | Title, Abstract |
|  | **INTRODUCTION** |  |  |  |
| 2 | **Background** | Explain the scientific background and rationale for the reported study. What is the exposure? Is a potential causal relationship between exposure and outcome plausible? Justify why MR is a helpful method to address the study question | 2 | Introduction: paragraphs 1-3 |
| 3 | **Objectives** | State specific objectives clearly, including pre-specified causal hypotheses (if any). State that MR is a method that, under specific assumptions, intends to estimate causal effects | 2 | Introduction: paragraphs 1-3 |
|  | **METHODS** |  |  |  |
| 4 | **Study design and data sources** | Present key elements of the study design early in the article. Consider including a table listing sources of data for all phases of the study. For each data source contributing to the analysis, describe the following: |  |  |
|  | a) | Setting: Describe the study design and the underlying population, if possible. Describe the setting, locations, and relevant dates, including periods of recruitment, exposure, follow-up, and data collection, when available. | 3 | Material and Method: Study design |
|  | b) | Participants: Give the eligibility criteria, and the sources and methods of selection of participants. Report the sample size, and whether any power or sample size calculations were carried out prior to the main analysis | 4 | Material and Method: Data sources, Table1 |
|  | c) | Describe measurement, quality control and selection of genetic variants | 4 | Material and Method: Selection of Genetic Variants |
|  | d) | For each exposure, outcome, and other relevant variables, describe methods of assessment and diagnostic criteria for diseases | 4 | Material and Method: Data sources, Table1 |
|  | e) | Provide details of ethics committee approval and participant informed consent, if relevant | 6 | Material and Method: Ethnic |
| 5 | **Assumptions** | Explicitly state the three core IV assumptions for the main analysis (relevance, independence and exclusion restriction) as well assumptions for any additional or sensitivity analysis | 3-5 | Material and Method: Study design, Mendelian randomization analyses and Sensitivity analyses |
| 6 | **Statistical methods: main analysis** | Describe statistical methods and statistics used |  |  |
|  | a) | Describe how quantitative variables were handled in the analyses (i.e., scale, units, model) | 4 | Material and Method: Selection of Genetic Variants |
|  | b) | Describe how genetic variants were handled in the analyses and, if applicable, how their weights were selected | 4-5 | Material and Method: Selection of Genetic Variants, Mendelian randomization analyses |
|  | c) | Describe the MR estimator (e.g. two-stage least squares, Wald ratio) and related statistics. Detail the included covariates and, in case of two-sample MR, whether the same covariate set was used for adjustment in the two samples | 4-5 | Material and Method: Selection of Genetic Variants, Mendelian randomization analyses |
|  | d) | Explain how missing data were addressed |  | Not applicable |
|  | e) | If applicable, indicate how multiple testing was addressed |  | Not applicable |
| 7 | **Assessment of assumptions** | Describe any methods or prior knowledge used to assess the assumptions or justify their validity |  |  |
| 8 | **Sensitivity analyses and additional analyses** | Describe any sensitivity analyses or additional analyses performed (e.g. comparison of effect estimates from different approaches, independent replication, bias analytic techniques, validation of instruments, simulations) | 3-5 | Material and Method: Study design, Mendelian randomization analyses and Sensitivity analyses |
| 9 | **Software and pre-registration** |  |  |  |
|  | a) | Name statistical software and package(s), including version and settings used | 6 | Material and Method: Software and packages |
|  | b) | State whether the study protocol and details were pre-registered (as well as when and where) |  | Not applicable |
|  | **RESULTS** |  |  |  |
| 10 | **Descriptive data** |  |  |  |
|  | a) | Report the numbers of individuals at each stage of included studies and reasons for exclusion. Consider use of a flow diagram | 3 | Material and Method: Study design, Table1 |
|  | b) | Report summary statistics for phenotypic exposure(s), outcome(s), and other relevant variables (e.g. means, SDs, proportions) | 4 | Material and Method: Data sources, Table s2 |
|  | c) | If the data sources include meta-analyses of previous studies, provide the assessments of heterogeneity across these studies |  | Not applicable |
|  | d) | For two-sample MR:  i.  Provide justification of the similarity of the genetic variant-exposure associations between the exposure and outcome samples  ii.  Provide information on the number of individuals who overlap between the exposure and outcome studies | 3-4 | Material and Method: Study design and Data sources, Table1 |
| 11 | **Main results** |  |  |  |
|  | a) | Report the associations between genetic variant and exposure, and between genetic variant and outcome, preferably on an interpretable scale | 7 | Result: Mendelian randomization: Selection of Genetic Variants and Table S2 |
|  | b) | Report MR estimates of the relationship between exposure and outcome, and the measures of uncertainty from the MR analysis, on an interpretable scale, such as odds ratio or relative risk per SD difference | 7-8 | Result: Mendelian randomization: Effect of autoimmune disorders on vitiligo, Effect of vitiligo on autoimmune disorders and Figure 1-2 |
|  | c) | If relevant, consider translating estimates of relative risk into absolute risk for a meaningful time period |  | Not applicable |
|  | d) | Consider plots to visualize results (e.g. forest plot, scatterplot of associations between genetic variants and outcome versus between genetic variants and exposure) |  | Figure 1-2 |
| 12 | **Assessment of assumptions** |  |  |  |
|  | a) | Report the assessment of the validity of the assumptions | 8 | Result: Mendelian randomization: Sensitivity analysis and Table s3-4 |
|  | b) | Report any additional statistics (e.g., assessments of heterogeneity across genetic variants, such as *I^2^*, Q statistic or E-value) | 8 | Result: Mendelian randomization: Sensitivity analysis and Table s3-4 |
| 13 | **Sensitivity analyses and additional analyses** |  |  |  |
|  | a) | Report any sensitivity analyses to assess the robustness of the main results to violations of the assumptions | 8 | Result: Mendelian randomization: Sensitivity analysis and Table s3-4 |
|  | b) | Report results from other sensitivity analyses or additional analyses | 7-8 | Result: Mendelian randomization: Effect of autoimmune disorders on vitiligo, Effect of vitiligo on autoimmune disorders and Figure 1-2 |
|  | c) | Report any assessment of direction of causal relationship (e.g., bidirectional MR) | 7-8 | Result: Mendelian randomization: Effect of autoimmune disorders on vitiligo, Effect of vitiligo on autoimmune disorders and Figure 1-2 |
|  | d) | When relevant, report and compare with estimates from non-MR analyses |  | Not applicable |
|  | e) | Consider additional plots to visualize results (e.g., leave-one-out analyses) |  | Figure s3 |
|  | **DISCUSSION** |  |  |  |
| 14 | **Key results** | Summarize key results with reference to study objectives | 9 | Discussion:paragraph 1 |
| 15 | **Limitations** | Discuss limitations of the study, taking into account the validity of the IV assumptions, other sources of potential bias, and imprecision. Discuss both direction and magnitude of any potential bias and any efforts to address them |  | Not applicable |
| 16 | **Interpretation** |  |  |  |
|  | a) | Meaning: Give a cautious overall interpretation of results in the context of their limitations and in comparison with other studies | 9-10 | Discussion:paragraph 2-5 |
|  | b) | Mechanism: Discuss underlying biological mechanisms that could drive a potential causal relationship between the investigated exposure and the outcome, and whether the gene-environment equivalence assumption is reasonable. Use causal language carefully, clarifying that IV estimates may provide causal effects only under certain assumptions | 9-10 | Discussion:paragraph 2-5 |
|  | c) | Clinical relevance: Discuss whether the results have clinical or public policy relevance, and to what extent they inform effect sizes of possible interventions | 9-10 | Discussion:paragraph 2-5 |
| 17 | **Generalizability** | Discuss the generalizability of the study results (a) to other populations, (b) across other exposure periods/timings, and (c) across other levels of exposure | 9 | Discussion:paragraph 2 |
|  | **OTHER INFORMATION** |  |  |  |
| 18 | **Funding** | Describe sources of funding and the role of funders in the present study and, if applicable, sources of funding for the databases and original study or studies on which the present study is based | 11 | Funding |
| 19 | **Data and data sharing** | Provide the data used to perform all analyses or report where and how the data can be accessed, and reference these sources in the article. Provide the statistical code needed to reproduce the results in the article, or report whether the code is publicly accessible and if so, where | 11 | Data availability statement |
| 20 | **Conflicts of Interest** | All authors should declare all potential conflicts of interest | 11 | Conflict of interest |

**Table S2. Overview of the IVs utilized in the bidirectional MR analyses**

| **Trait** | **SNP** | **P-value** | **Effect allele** | **Other allele** | **Beta** | **SE** | **F** |
| --- | --- | --- | --- | --- | --- | --- | --- |
| **Vitiligo** | rs6679677 | 1.83E-16 | A | C | 0.32930375 | 0.04 | 67.8 |
|  | rs159960 | 5.18E-12 | A | G | 0.20701417 | 0.03 | 47.6 |
|  | rs10798176 | 1.02E-13 | C | T | 0.22314355 | 0.03 | 55.3 |
|  | rs16843742 | 1.38E-07 | C | T | -0.210721 | 0.04 | 27.8 |
|  | rs17047094 | 4.48E-15 | G | A | 0.39204209 | 0.05 | 61.5 |
|  | rs62160391 | 1.66E-07 | A | C | 0.15700375 | 0.03 | 27.4 |
|  | rs2111485 | 8.86E-22 | A | G | -0.2876821 | 0.03 | 92 |
|  | rs3096849 | 6.69E-09 | G | A | 0.17395331 | 0.03 | 33.6 |
|  | rs62246015 | 1.02E-13 | T | C | -0.2231436 | 0.03 | 55.3 |
|  | rs11535078 | 1.83E-16 | T | A | 0.32930375 | 0.04 | 67.8 |
|  | rs2037184 | 6.18E-09 | C | T | -0.1743534 | 0.03 | 33.8 |
|  | rs13076312 | 2.15E-20 | T | C | 0.27763174 | 0.03 | 85.6 |
|  | rs4078470 | 3.39E-11 | A | T | 0.19885086 | 0.03 | 43.9 |
|  | rs1632908 | 1.11E-17 | G | C | -0.3424903 | 0.04 | 73.3 |
|  | rs28752490 | 1.60E-71 | A | G | 0.53649337 | 0.03 | 320 |
|  | rs28688825 | 2.78E-30 | G | A | 0.45742485 | 0.04 | 131 |
|  | rs12212737 | 1.22E-09 | A | G | 0.18232156 | 0.03 | 36.9 |
|  | rs2247315 | 3.92E-15 | C | A | -0.2357223 | 0.03 | 61.7 |
|  | rs117744081 | 5.82E-21 | G | A | 0.65752 | 0.07 | 88.2 |
|  | rs2687834 | 2.10E-10 | G | T | 0.19062036 | 0.03 | 40.4 |
|  | rs7007905 | 5.27E-10 | G | C | -0.1863296 | 0.03 | 38.6 |
|  | rs706779 | 1.05E-23 | C | T | -0.3011051 | 0.03 | 101 |
|  | rs7893324 | 3.79E-09 | C | T | -0.2357223 | 0.04 | 34.7 |
|  | rs3814231 | 5.27E-10 | T | C | -0.1863296 | 0.03 | 38.6 |
|  | rs932635 | 5.18E-12 | A | G | 0.20701417 | 0.03 | 47.6 |
|  | rs1126809 | 1.26E-43 | A | G | -0.4155154 | 0.03 | 192 |
|  | rs11021232 | 6.89E-27 | C | T | 0.3220835 | 0.03 | 115 |
|  | rs583642 | 6.69E-09 | T | C | 0.17395331 | 0.03 | 33.6 |
|  | rs1689510 | 2.24E-19 | C | G | 0.27002714 | 0.03 | 81 |
|  | rs10774624 | 1.02E-13 | A | G | -0.2231436 | 0.03 | 55.3 |
|  | rs111260018 | 1.13E-08 | A | G | 0.57097955 | 0.1 | 32.6 |
|  | rs6573910 | 5.18E-12 | T | C | 0.20701417 | 0.03 | 47.6 |
|  | rs12904397 | 3.03E-13 | C | G | 0.36464311 | 0.05 | 53.2 |
|  | rs4268748 | 9.57E-26 | C | T | -0.3147107 | 0.03 | 110 |
|  | rs8083511 | 5.18E-12 | C | A | 0.20701417 | 0.03 | 47.6 |
|  | rs6510827 | 6.69E-09 | T | C | 0.17395331 | 0.03 | 33.6 |
|  | rs2082481 | 2.16E-12 | A | G | -0.210721 | 0.03 | 49.3 |
|  | rs62211989 | 1.35E-14 | C | G | -0.4620355 | 0.06 | 59.3 |
|  | rs111462340 | 1.46E-31 | A | G | 0.35065687 | 0.03 | 137 |
|  | rs229527 | 1.74E-22 | A | C | 0.29266961 | 0.03 | 95.2 |
|  | rs5751086 | 3.92E-15 | T | C | -0.2357223 | 0.03 | 61.7 |
| **SLE** | rs6679677 | 4.55E-13 | A | C | 0.336472 | 0.046 | 52.4 |
|  | rs4661543 | 9.40E-11 | G | T | 0.274437 | 0.042 | 41.9 |
|  | rs10912578 | 1.65E-15 | G | A | -0.24686 | 0.031 | 63.4 |
|  | rs17849501 | 1.81E-59 | T | C | 0.81093 | 0.05 | 264 |
|  | rs6671847 | 6.64E-12 | A | G | 0.198851 | 0.029 | 47.1 |
|  | rs4916215 | 5.07E-11 | T | C | 0.223144 | 0.034 | 43.2 |
|  | rs12094036 | 1.37E-08 | C | T | -0.328504 | 0.058 | 32.2 |
|  | rs13019891 | 1.65E-83 | T | G | -0.562119 | 0.029 | 375 |
|  | rs2573219 | 1.13E-42 | C | A | 0.587787 | 0.043 | 187 |
|  | rs10200680 | 4.96E-09 | T | C | -0.248461 | 0.042 | 34.2 |
|  | rs268124 | 8.60E-09 | T | C | 0.18633 | 0.032 | 33.1 |
|  | rs2459611 | 7.62E-09 | T | C | 0.261365 | 0.045 | 33.4 |
|  | rs4274624 | 9.73E-66 | T | C | -0.559616 | 0.033 | 293 |
|  | rs10048743 | 2.04E-08 | T | G | -0.231112 | 0.041 | 31.5 |
|  | rs34703115 | 4.08E-09 | C | T | -0.616186 | 0.105 | 34.6 |
|  | rs1464446 | 2.79E-16 | T | G | -0.328504 | 0.04 | 66.9 |
|  | rs9852014 | 2.26E-36 | G | A | 0.620577 | 0.049 | 159 |
|  | rs13136219 | 3.50E-10 | T | C | -0.174353 | 0.028 | 39.4 |
|  | rs1078324 | 7.11E-20 | A | C | -0.71335 | 0.078 | 83.3 |
|  | rs4388254 | 3.71E-10 | T | C | 0.378436 | 0.06 | 39.3 |
|  | rs2431697 | 2.60E-14 | C | T | -0.223144 | 0.029 | 58 |
|  | rs6889239 | 2.19E-18 | C | T | 0.277632 | 0.032 | 76.5 |
|  | rs389884 | 2.92E-102 | G | A | 0.928219 | 0.043 | 461 |
|  | rs9274357 | 1.28E-38 | T | C | 0.457425 | 0.035 | 169 |
|  | rs7768653 | 3.11E-12 | T | C | -0.207014 | 0.03 | 48.6 |
|  | rs12524498 | 2.48E-08 | T | G | -0.673345 | 0.121 | 31.1 |
|  | rs58721818 | 3.38E-18 | T | C | 0.65752 | 0.076 | 75.7 |
|  | rs150180633 | 2.66E-41 | T | C | 0.928219 | 0.069 | 181 |
|  | rs35000415 | 1.86E-45 | T | C | 0.587787 | 0.042 | 200 |
|  | rs2736332 | 4.83E-18 | C | G | 0.277632 | 0.032 | 74.9 |
|  | rs7823055 | 1.64E-34 | T | G | -0.350657 | 0.029 | 150 |
|  | rs7899626 | 4.19E-08 | T | C | 0.182322 | 0.033 | 30.1 |
|  | rs7097397 | 8.60E-11 | A | G | -0.18633 | 0.029 | 42.1 |
|  | rs58688157 | 2.97E-11 | G | A | -0.223144 | 0.034 | 44.2 |
|  | rs353608 | 2.93E-11 | G | A | 0.18633 | 0.028 | 44.2 |
|  | rs73050535 | 9.11E-09 | T | C | -0.71335 | 0.124 | 33 |
|  | rs597808 | 3.51E-08 | G | A | -0.162519 | 0.029 | 30.4 |
|  | rs1143679 | 5.03E-48 | A | G | 0.582216 | 0.04 | 212 |
|  | rs13332649 | 5.43E-17 | G | A | -0.314711 | 0.038 | 70.2 |
|  | rs143123127 | 2.23E-08 | A | G | 0.470004 | 0.084 | 31.3 |
|  | rs35251378 | 3.61E-13 | A | G | -0.235722 | 0.032 | 52.8 |
|  | rs73068668 | 4.40E-08 | A | G | -0.314711 | 0.057 | 30 |
|  | rs3747093 | 2.88E-14 | A | G | 0.262364 | 0.035 | 57.8 |
| **Graves' disease** | rs12745574 | 1.85E-06 | A | G | 1.22247 | 0.256 | 22.7 |
|  | rs6722414 | 2.04E-06 | A | G | 0.660114 | 0.139 | 22.6 |
|  | rs1683546 | 8.78E-06 | C | T | 0.836679 | 0.188 | 19.8 |
|  | rs68057871 | 2.70E-06 | T | G | 0.583657 | 0.124 | 22 |
|  | rs13020919 | 5.28E-06 | T | C | 0.493424 | 0.108 | 20.7 |
|  | rs12633624 | 5.68E-06 | T | A | 1.60227 | 0.353 | 20.6 |
|  | rs77685804 | 4.19E-06 | T | C | 1.64751 | 0.358 | 21.2 |
|  | rs140182038 | 3.83E-07 | T | C | 1.9266 | 0.379 | 25.8 |
|  | rs17071071 | 5.83E-06 | C | A | 2.29413 | 0.506 | 20.5 |
|  | rs9270868 | 3.82E-13 | G | A | 0.9493 | 0.131 | 52.7 |
|  | rs2785901 | 1.38E-06 | T | C | 2.27095 | 0.47 | 23.3 |
|  | rs74699117 | 9.60E-06 | A | G | 1.32853 | 0.3 | 19.6 |
|  | rs12707540 | 7.27E-06 | C | A | 0.442256 | 0.099 | 20.1 |
|  | rs553195049 | 6.64E-06 | T | C | 1.52742 | 0.339 | 20.3 |
|  | rs145533960 | 8.48E-06 | G | A | 3.31873 | 0.745 | 19.8 |
|  | rs28868288 | 8.44E-07 | G | C | 0.931351 | 0.189 | 24.3 |
|  | rs187910662 | 7.34E-07 | G | A | 2.64541 | 0.534 | 24.5 |
|  | rs4936947 | 9.37E-06 | T | C | -0.530716 | 0.12 | 19.6 |
|  | rs113052666 | 9.49E-06 | C | T | 0.761301 | 0.172 | 19.6 |
|  | rs72651010 | 5.70E-06 | T | C | 1.70935 | 0.377 | 20.6 |
|  | rs35203278 | 6.87E-06 | C | T | 0.539516 | 0.12 | 20.2 |
|  | rs16940155 | 9.43E-06 | T | C | 1.39744 | 0.315 | 19.6 |
|  | rs2911435 | 7.29E-06 | T | C | 1.6172 | 0.361 | 20.1 |
|  | rs116937920 | 9.06E-06 | A | G | 1.27175 | 0.287 | 19.7 |
|  | rs113781412 | 1.33E-06 | A | G | 1.93953 | 0.401 | 23.4 |
|  | rs2837919 | 8.00E-06 | G | A | 0.592205 | 0.133 | 19.9 |
| **IBD** | rs12103 | 3.28E-11 | A | G | 0.0867354 | 0.013 | 44 |
|  | rs72634258 | 1.25E-19 | A | G | 0.126877 | 0.014 | 82.2 |
|  | rs7523442 | 2.76E-36 | A | G | 0.124537 | 0.01 | 158 |
|  | rs6588248 | 1.38E-16 | A | C | -0.0819867 | 0.01 | 68.3 |
|  | rs7547569 | 1.65E-170 | A | G | 0.6472 | 0.023 | 775 |
|  | rs34856868 | 9.80E-09 | A | G | -0.195275 | 0.034 | 32.9 |
|  | rs2974935 | 8.87E-12 | A | C | 0.0687256 | 0.01 | 46.6 |
|  | rs2297559 | 1.89E-11 | A | G | 0.0741659 | 0.011 | 45.1 |
|  | rs10800309 | 6.16E-37 | A | G | 0.132111 | 0.01 | 161 |
|  | rs12411259 | 6.18E-09 | A | G | 0.0669054 | 0.012 | 33.8 |
|  | rs2488397 | 4.55E-16 | C | G | 0.09881 | 0.012 | 66 |
|  | rs35730213 | 8.33E-45 | C | G | -0.159624 | 0.011 | 197 |
|  | rs3024493 | 1.65E-50 | A | C | 0.196922 | 0.013 | 223 |
|  | rs13407913 | 1.69E-20 | A | G | -0.0917072 | 0.01 | 86.1 |
|  | rs780094 | 3.88E-15 | A | G | 0.0783055 | 0.01 | 61.8 |
|  | rs78487399 | 7.71E-16 | C | G | -0.132093 | 0.016 | 64.9 |
|  | rs7608910 | 2.60E-36 | A | G | -0.126444 | 0.01 | 158 |
|  | rs6740462 | 5.59E-12 | A | C | 0.0799597 | 0.012 | 47.5 |
|  | rs1420098 | 1.82E-20 | A | G | 0.0952951 | 0.01 | 86 |
|  | rs1990760 | 3.56E-10 | A | G | -0.0671151 | 0.011 | 39.3 |
|  | rs1517352 | 3.87E-14 | A | C | -0.0778816 | 0.01 | 57.2 |
|  | rs72924296 | 1.44E-08 | A | G | 0.0638392 | 0.011 | 32.1 |
|  | rs11677953 | 2.92E-15 | A | G | 0.0790564 | 0.01 | 62.3 |
|  | rs35256947 | 3.87E-13 | A | G | -0.0821647 | 0.011 | 52.7 |
|  | rs6708373 | 1.43E-41 | A | G | -0.134178 | 0.01 | 182 |
|  | rs6745185 | 1.37E-09 | A | C | -0.0698267 | 0.012 | 36.7 |
|  | rs11713774 | 3.92E-11 | A | G | -0.0942858 | 0.014 | 43.7 |
|  | rs9836291 | 9.61E-53 | A | G | 0.160867 | 0.011 | 234 |
|  | rs4692386 | 1.21E-08 | A | G | -0.0579752 | 0.01 | 32.5 |
|  | rs13107612 | 1.62E-11 | A | G | 0.0732561 | 0.011 | 45.4 |
|  | rs974801 | 7.07E-13 | A | G | 0.0727718 | 0.01 | 51.5 |
|  | rs7657746 | 1.83E-13 | A | G | 0.0868542 | 0.012 | 54.2 |
|  | rs3776414 | 2.65E-14 | A | C | -0.0773716 | 0.01 | 58 |
|  | rs7711427 | 4.63E-66 | A | C | -0.174764 | 0.01 | 295 |
|  | rs79980175 | 1.30E-10 | A | C | 0.0953162 | 0.015 | 41.3 |
|  | rs4703855 | 7.16E-11 | A | G | -0.0710611 | 0.011 | 42.5 |
|  | rs34804116 | 3.62E-08 | A | C | -0.0574631 | 0.01 | 30.3 |
|  | rs272882 | 1.47E-52 | A | C | 0.166117 | 0.011 | 233 |
|  | rs181826 | 4.05E-15 | A | C | 0.0820444 | 0.01 | 61.7 |
|  | rs71593329 | 1.19E-14 | A | C | 0.0978042 | 0.013 | 59.6 |
|  | rs36048684 | 3.70E-09 | A | T | -0.0941465 | 0.016 | 34.8 |
|  | rs56167332 | 7.17E-50 | A | C | 0.155855 | 0.01 | 220 |
|  | rs4976646 | 3.23E-12 | A | G | -0.0730113 | 0.01 | 48.5 |
|  | rs7773324 | 5.84E-09 | A | G | 0.061818 | 0.011 | 33.9 |
|  | rs1267499 | 5.22E-11 | A | G | -0.0821443 | 0.013 | 43.1 |
|  | rs2328546 | 1.30E-13 | A | G | -0.0940162 | 0.013 | 54.9 |
|  | rs9264942 | 1.55E-18 | A | G | -0.094692 | 0.011 | 77.2 |
|  | rs9273363 | 3.30E-58 | A | C | -0.193129 | 0.012 | 259 |
|  | rs1847472 | 6.63E-10 | A | C | -0.0672805 | 0.011 | 38.1 |
|  | rs11152949 | 7.25E-23 | A | G | -0.105057 | 0.011 | 96.9 |
|  | rs11758694 | 1.75E-11 | A | T | 0.107782 | 0.016 | 45.2 |
|  | rs6933404 | 5.84E-15 | A | G | -0.0957518 | 0.012 | 61 |
|  | rs62434177 | 1.14E-08 | A | G | -0.179105 | 0.031 | 32.6 |
|  | rs9457247 | 2.48E-18 | A | G | 0.089151 | 0.01 | 76.3 |
|  | rs1182188 | 1.08E-09 | A | G | 0.0659028 | 0.011 | 37.2 |
|  | rs4917129 | 9.48E-15 | A | G | -0.0794435 | 0.01 | 60 |
|  | rs2395022 | 8.27E-15 | A | C | 0.181635 | 0.023 | 60.3 |
|  | rs6466198 | 2.18E-16 | A | T | -0.0841312 | 0.01 | 67.4 |
|  | rs2538470 | 3.00E-11 | A | G | 0.0675599 | 0.01 | 44.2 |
|  | rs7011507 | 2.03E-08 | A | G | -0.0846011 | 0.015 | 31.5 |
|  | rs7015630 | 2.90E-08 | A | G | 0.0627799 | 0.011 | 30.8 |
|  | rs10956252 | 2.26E-16 | C | G | -0.0834795 | 0.01 | 67.4 |
|  | rs6651252 | 9.08E-10 | A | G | 0.0908484 | 0.015 | 37.5 |
|  | rs10758669 | 4.70E-48 | A | C | -0.148762 | 0.01 | 212 |
|  | rs4743820 | 3.80E-09 | A | G | 0.0639523 | 0.011 | 34.7 |
|  | rs7848647 | 3.16E-35 | A | G | -0.13239 | 0.011 | 153 |
|  | rs11793497 | 1.71E-54 | A | G | -0.156206 | 0.01 | 242 |
|  | rs12722515 | 4.57E-12 | A | C | -0.0989022 | 0.014 | 47.9 |
|  | rs2050392 | 1.87E-11 | A | G | 0.0691178 | 0.01 | 45.1 |
|  | rs34779708 | 2.07E-25 | A | C | -0.106679 | 0.01 | 109 |
|  | rs2153283 | 1.54E-11 | A | C | -0.0859637 | 0.013 | 45.5 |
|  | rs10761659 | 4.97E-53 | A | G | -0.153811 | 0.01 | 235 |
|  | rs2688608 | 2.75E-10 | A | C | 0.062403 | 0.01 | 39.8 |
|  | rs1250566 | 4.77E-20 | A | G | -0.100894 | 0.011 | 84.1 |
|  | rs2497318 | 1.36E-10 | A | G | -0.0635464 | 0.01 | 41.2 |
|  | rs10748781 | 2.28E-63 | A | C | -0.169179 | 0.01 | 282 |
|  | rs2274351 | 6.93E-09 | A | G | 0.0604993 | 0.01 | 33.6 |
|  | rs12796489 | 2.87E-69 | A | C | -0.760367 | 0.043 | 309 |
|  | rs11230563 | 1.71E-14 | A | G | -0.081194 | 0.011 | 58.8 |
|  | rs559928 | 3.33E-13 | A | G | -0.094388 | 0.013 | 53 |
|  | rs11236797 | 9.32E-52 | A | C | 0.150864 | 0.01 | 229 |
|  | rs648541 | 1.22E-09 | A | G | 0.0648616 | 0.011 | 36.9 |
|  | rs1388585 | 6.85E-22 | A | G | -0.30489 | 0.032 | 92.5 |
|  | rs10878302 | 5.26E-09 | A | T | 0.112429 | 0.019 | 34.1 |
|  | rs12318183 | 1.67E-27 | A | C | 0.109531 | 0.01 | 118 |
|  | rs3184504 | 1.29E-09 | A | G | 0.0600317 | 0.01 | 36.8 |
|  | rs12585310 | 5.25E-11 | A | G | 0.0706485 | 0.011 | 43.1 |
|  | rs941823 | 6.19E-13 | A | G | -0.0830172 | 0.012 | 51.8 |
|  | rs6561151 | 3.53E-17 | A | G | 0.1 | 0.012 | 71 |
|  | rs9557207 | 3.52E-13 | A | G | 0.0878448 | 0.012 | 52.9 |
|  | rs10142466 | 1.08E-08 | A | G | 0.0580054 | 0.01 | 32.7 |
|  | rs1569328 | 3.21E-09 | A | G | -0.0809722 | 0.014 | 35.1 |
|  | rs55808324 | 5.14E-17 | A | G | 0.141213 | 0.017 | 70.3 |
|  | rs17651741 | 2.81E-08 | A | G | 0.0702476 | 0.013 | 30.8 |
|  | rs17293632 | 2.71E-20 | A | G | 0.107165 | 0.012 | 85.2 |
|  | rs367569 | 1.93E-17 | A | G | -0.0958166 | 0.011 | 72.2 |
|  | rs62037363 | 6.36E-22 | A | G | -0.0987549 | 0.01 | 92.6 |
|  | rs6500315 | 1.12E-10 | A | G | -0.0766082 | 0.012 | 41.6 |
|  | rs7194886 | 2.53E-36 | A | G | -0.126026 | 0.01 | 158 |
|  | rs2270395 | 5.17E-11 | A | G | 0.0778411 | 0.012 | 43.1 |
|  | rs11641016 | 9.51E-17 | C | G | 0.111288 | 0.013 | 69.1 |
|  | rs144004051 | 4.16E-08 | A | G | -0.214846 | 0.039 | 30.1 |
|  | rs9889296 | 1.35E-20 | A | G | -0.104999 | 0.011 | 86.6 |
|  | rs4795397 | 8.30E-44 | A | G | -0.138343 | 0.01 | 193 |
|  | rs744166 | 1.14E-22 | A | G | 0.100017 | 0.01 | 96 |
|  | rs1292053 | 9.89E-13 | A | G | -0.0701035 | 0.01 | 50.9 |
|  | rs17780256 | 3.19E-11 | A | C | 0.083427 | 0.013 | 44.1 |
|  | rs2847278 | 8.33E-28 | A | G | -0.144528 | 0.013 | 119 |
|  | rs7240004 | 1.01E-10 | A | G | 0.0665215 | 0.01 | 41.8 |
|  | rs2024092 | 1.12E-18 | A | G | 0.106813 | 0.012 | 77.8 |
|  | rs35164067 | 2.66E-20 | A | G | -0.11754 | 0.013 | 85.2 |
|  | rs17694108 | 1.21E-14 | A | G | 0.0857629 | 0.011 | 59.5 |
|  | rs516246 | 1.15E-13 | A | G | 0.0755599 | 0.01 | 55.1 |
|  | rs6058869 | 2.63E-08 | A | G | 0.0556615 | 0.01 | 31 |
|  | rs6074022 | 8.32E-11 | A | G | -0.0742587 | 0.011 | 42.2 |
|  | rs913678 | 5.35E-11 | A | G | 0.0691643 | 0.011 | 43 |
|  | rs259964 | 6.93E-12 | A | G | 0.0674584 | 0.01 | 47 |
|  | rs6062496 | 2.11E-33 | A | G | 0.123216 | 0.01 | 145 |
|  | rs1297258 | 5.38E-30 | A | G | -0.114524 | 0.01 | 129 |
|  | rs2836883 | 3.38E-48 | A | G | -0.168413 | 0.012 | 213 |
|  | rs8127691 | 8.98E-30 | A | G | 0.114259 | 0.01 | 128 |
|  | rs2266961 | 2.56E-13 | C | G | 0.0906694 | 0.012 | 53.5 |
|  | rs1003342 | 3.30E-15 | A | G | 0.0801818 | 0.01 | 62.1 |
|  | rs2143178 | 4.80E-38 | A | G | 0.176684 | 0.014 | 166 |
|  | rs11691685 | 7.27E-11 | A | G | 0.122467 | 0.019 | 42.4 |
|  | rs1363907 | 4.87E-15 | A | G | 0.0815026 | 0.01 | 61.3 |
|  | rs769177 | 6.53E-20 | A | G | 0.260903 | 0.029 | 83.5 |
|  | rs13204742 | 5.39E-10 | A | C | 0.0916208 | 0.015 | 38.5 |
|  | rs3801835 | 1.47E-09 | A | G | 0.064107 | 0.011 | 36.6 |
|  | rs7253253 | 6.19E-09 | A | C | -0.134424 | 0.023 | 33.8 |
|  | rs6456426 | 8.18E-11 | A | C | -0.0643405 | 0.01 | 42.2 |
|  | rs67643815 | 6.42E-10 | A | C | -0.0629092 | 0.01 | 38.2 |
|  | rs6111031 | 1.23E-71 | A | G | -0.264091 | 0.015 | 320 |
| **Alopecia areata** | rs17108270 | 3.80E-06 | A | T | -0.293679 | 0.064 | 21.4 |
|  | rs188947455 | 8.65E-06 | A | G | 1.18363 | 0.266 | 19.8 |
|  | rs191875864 | 5.37E-06 | T | C | 0.426549 | 0.094 | 20.7 |
|  | rs4662987 | 9.20E-06 | C | T | 0.531303 | 0.12 | 19.7 |
|  | rs73030033 | 7.67E-07 | C | T | 0.851854 | 0.172 | 24.4 |
|  | rs112074564 | 8.99E-06 | T | C | 2.18615 | 0.492 | 19.7 |
|  | rs9273060 | 5.11E-06 | T | A | 0.269267 | 0.059 | 20.8 |
|  | rs9275563 | 4.31E-12 | T | C | 0.377003 | 0.054 | 48 |
|  | rs74763871 | 5.90E-06 | T | C | 0.521148 | 0.115 | 20.5 |
|  | rs427 | 2.58E-06 | A | G | 0.279908 | 0.06 | 22.1 |
|  | rs1361957 | 9.94E-06 | G | A | -0.237428 | 0.054 | 19.5 |
|  | rs111502065 | 7.11E-06 | A | G | -1.20983 | 0.269 | 20.2 |
|  | rs4870917 | 9.75E-06 | C | G | 0.396509 | 0.09 | 19.6 |
|  | rs142810656 | 2.80E-06 | T | G | 0.355258 | 0.076 | 22 |
|  | rs56330738 | 8.16E-06 | T | C | -0.315958 | 0.071 | 19.9 |
|  | rs28637800 | 3.12E-06 | A | G | 0.436587 | 0.094 | 21.7 |
|  | rs148578443 | 3.50E-06 | G | C | 1.54963 | 0.334 | 21.5 |
| **Type Ⅰ diabetes** | rs2269247 | 7.28E-09 | T | C | 0.1709 | 0.03 | 33.6 |
|  | rs6679677 | 3.42E-79 | A | C | 0.6527 | 0.035 | 356 |
|  | rs10911399 | 6.75E-09 | G | A | -0.3707 | 0.064 | 33.5 |
|  | rs11571297 | 1.11E-16 | C | T | -0.1964 | 0.024 | 68.7 |
|  | rs192324744 | 1.37E-10 | G | T | 0.562 | 0.088 | 41.3 |
|  | rs17863786 | 4.26E-11 | G | A | 0.4144 | 0.063 | 43.5 |
|  | rs10183097 | 1.82E-10 | C | T | 0.2053 | 0.032 | 40.7 |
|  | rs6719660 | 2.52E-08 | G | A | 0.2918 | 0.052 | 31 |
|  | rs10865468 | 4.66E-09 | C | G | -0.1624 | 0.028 | 34.4 |
|  | rs1869449 | 4.55E-11 | A | G | 0.1769 | 0.027 | 43.2 |
|  | rs2111485 | 1.89E-10 | G | A | 0.1577 | 0.025 | 40.4 |
|  | rs1027769 | 3.52E-10 | T | G | -0.9962 | 0.159 | 39.4 |
|  | rs62410259 | 1.02E-12 | A | G | -0.3796 | 0.053 | 50.7 |
|  | rs34954 | 1.25E-08 | A | C | -0.4912 | 0.086 | 32.4 |
|  | rs13182737 | 1.49E-08 | A | G | 0.1465 | 0.026 | 32 |
|  | rs9469220 | 1.01E-122 | A | G | -0.6892 | 0.029 | 553 |
|  | rs9468541 | 1.27E-16 | A | G | 0.3616 | 0.044 | 68.5 |
|  | rs2535319 | 1.67E-35 | C | T | 0.3495 | 0.028 | 155 |
|  | rs3998158 | 1.90E-159 | C | T | 0.8509 | 0.032 | 725 |
|  | rs111846582 | 9.98E-09 | A | G | 0.4628 | 0.081 | 32.8 |
|  | rs3129761 | 1.00E-200 | C | G | 1.2056 | 0.032 | 1411 |
|  | rs142379540 | 4.82E-16 | G | A | -0.6022 | 0.074 | 65.9 |
|  | rs34296259 | 1.43E-08 | A | T | 0.6637 | 0.117 | 32.1 |
|  | rs10760335 | 2.43E-08 | G | A | 0.1357 | 0.024 | 31.2 |
|  | rs12722495 | 1.27E-14 | C | T | -0.3145 | 0.041 | 59.4 |
|  | rs77523242 | 5.42E-09 | C | T | -0.3705 | 0.064 | 34 |
|  | rs10830227 | 1.02E-11 | A | G | 0.1582 | 0.023 | 46.1 |
|  | rs79075295 | 1.46E-11 | A | G | -0.4192 | 0.062 | 45.6 |
|  | rs689 | 2.30E-87 | T | A | 0.7004 | 0.035 | 391 |
|  | rs10774624 | 1.34E-25 | A | G | -0.2556 | 0.024 | 110 |
|  | rs1131017 | 4.24E-25 | G | C | -0.2461 | 0.024 | 107 |
|  | rs59680223 | 5.00E-10 | T | C | 0.6421 | 0.103 | 38.7 |
|  | rs61944737 | 8.85E-10 | G | A | 0.1819 | 0.03 | 37.5 |
|  | rs17125653 | 4.75E-09 | A | T | 0.2355 | 0.04 | 34.3 |
|  | rs55996894 | 3.13E-08 | C | G | -0.1785 | 0.032 | 30.5 |
|  | rs201417739 | 3.41E-10 | C | A | -0.416 | 0.066 | 39.4 |
|  | rs194749 | 5.37E-09 | C | T | -0.1638 | 0.028 | 34 |
|  | rs4566101 | 6.23E-12 | C | T | 0.1755 | 0.026 | 47.4 |
|  | rs231971 | 1.55E-09 | G | A | 0.2411 | 0.04 | 36.5 |
|  | rs741172 | 3.11E-15 | T | C | -0.2034 | 0.026 | 62.2 |
|  | rs8056814 | 1.99E-10 | A | G | 0.2641 | 0.042 | 40.5 |
|  | rs34536443 | 4.84E-10 | C | G | -0.4139 | 0.067 | 38.7 |
|  | rs202520 | 7.97E-10 | G | A | -0.1573 | 0.026 | 37.8 |
| **RA** | rs2793108 | 4.62E-08 | T | C | 0.0703 | 0.013 | 29.7 |
|  | rs706778 | 3.37E-16 | T | C | 0.1048 | 0.013 | 67 |
|  | rs77509998 | 6.84E-14 | T | G | 0.1269 | 0.017 | 56.4 |
|  | rs10905284 | 3.81E-11 | A | C | -0.0984 | 0.015 | 43.6 |
|  | rs4938573 | 1.39E-13 | T | C | 0.1221 | 0.017 | 54.8 |
|  | rs112438759 | 2.81E-08 | G | C | -0.28 | 0.05 | 30.9 |
|  | rs2476601 | 3.75E-168 | G | A | -0.5439 | 0.02 | 762 |
|  | rs12137270 | 2.20E-08 | T | C | 0.0846 | 0.015 | 31.4 |
|  | rs11810143 | 3.63E-09 | G | A | 0.1148 | 0.02 | 34.7 |
|  | rs4916340 | 1.52E-08 | T | G | 0.0836 | 0.015 | 31.9 |
|  | rs2240336 | 1.89E-10 | T | C | -0.0856 | 0.013 | 40.8 |
|  | rs3184504 | 1.04E-10 | C | T | -0.0873 | 0.014 | 41.8 |
|  | rs6429207 | 2.76E-09 | C | A | -0.1009 | 0.017 | 35.2 |
|  | rs867435 | 2.86E-11 | T | C | -0.0912 | 0.014 | 44.3 |
|  | rs1873914 | 4.44E-09 | C | G | -0.0762 | 0.013 | 34.4 |
|  | rs1696466 | 3.94E-08 | C | T | 0.0707 | 0.013 | 30 |
|  | rs9603608 | 2.32E-15 | C | A | -0.1087 | 0.014 | 63 |
|  | rs2045793 | 2.37E-10 | G | A | -0.0971 | 0.015 | 40.3 |
|  | rs7170151 | 9.49E-13 | T | C | 0.1022 | 0.014 | 51.1 |
|  | rs8026898 | 9.90E-23 | A | G | 0.1375 | 0.014 | 96.5 |
|  | rs115284761 | 2.78E-08 | C | T | -0.1166 | 0.021 | 30.8 |
|  | rs4584833 | 3.51E-10 | T | C | 0.084 | 0.013 | 39.3 |
|  | rs6659932 | 7.19E-09 | C | A | -0.1019 | 0.018 | 33.5 |
|  | rs9927316 | 2.57E-11 | G | C | 0.1084 | 0.016 | 44.8 |
|  | rs62067029 | 2.71E-10 | T | A | 0.0832 | 0.013 | 39.7 |
|  | rs641085 | 5.70E-12 | T | A | -0.0877 | 0.013 | 47.7 |
|  | rs75231016 | 2.94E-08 | C | G | -0.1465 | 0.026 | 30.8 |
|  | rs34536443 | 2.18E-20 | C | G | -0.44 | 0.048 | 85.4 |
|  | rs4239702 | 5.98E-13 | C | T | 0.1036 | 0.014 | 51.8 |
|  | rs12474386 | 1.03E-14 | A | G | -0.1 | 0.013 | 60.1 |
|  | rs8133843 | 1.33E-09 | A | G | 0.0851 | 0.014 | 36.9 |
|  | rs4853458 | 9.84E-17 | G | A | -0.1236 | 0.015 | 68.8 |
|  | rs3087243 | 2.72E-21 | A | G | -0.1208 | 0.013 | 89.1 |
|  | rs11089637 | 2.12E-10 | C | T | 0.111 | 0.018 | 40.2 |
|  | rs137687 | 7.79E-12 | A | G | -0.0893 | 0.013 | 47.2 |
|  | rs10173253 | 1.32E-08 | A | G | 0.0783 | 0.014 | 32.2 |
|  | rs34695944 | 1.03E-15 | C | T | 0.1036 | 0.013 | 64.5 |
|  | rs17534670 | 9.80E-13 | A | G | -0.0904 | 0.013 | 50.7 |
|  | rs9826420 | 2.16E-09 | C | G | 0.3252 | 0.054 | 35.9 |
|  | rs5019428 | 4.60E-12 | A | G | 0.0943 | 0.014 | 48.1 |
|  | rs9310852 | 1.08E-09 | G | A | 0.0791 | 0.013 | 37 |
|  | rs12506688 | 3.71E-22 | T | C | 0.1341 | 0.014 | 93.1 |
|  | rs2664035 | 3.85E-10 | A | G | 0.0815 | 0.013 | 39.3 |
|  | rs2561477 | 4.96E-11 | A | G | -0.0915 | 0.014 | 43.3 |
|  | rs5745271 | 1.14E-08 | G | T | 0.0749 | 0.013 | 32.7 |
|  | rs7731626 | 4.20E-33 | A | G | -0.186 | 0.016 | 144 |
|  | rs548234 | 7.30E-09 | T | C | -0.077 | 0.013 | 33.5 |
|  | rs17264332 | 4.14E-29 | G | A | 0.1684 | 0.015 | 126 |
|  | rs6570194 | 5.53E-09 | C | A | 0.1369 | 0.024 | 33.9 |
|  | rs113532504 | 1.61E-08 | T | C | 0.1215 | 0.022 | 31.9 |
|  | rs2451279 | 1.84E-10 | G | A | 0.0856 | 0.013 | 40.8 |
|  | rs3093017 | 7.72E-19 | G | C | -0.1155 | 0.013 | 78.9 |
|  | rs143182422 | 8.75E-09 | T | C | 0.1785 | 0.031 | 33.2 |
|  | rs3132662 | 1.26E-42 | A | G | 0.1729 | 0.013 | 188 |
|  | rs111455094 | 2.08E-11 | C | T | 0.2651 | 0.04 | 44.8 |
|  | rs4151671 | 8.34E-32 | T | C | 0.3387 | 0.029 | 137 |
|  | rs436845 | 2.42E-112 | G | A | -0.3465 | 0.015 | 506 |
|  | rs3129871 | 1.00E-200 | C | A | 0.5618 | 0.015 | 1481 |
|  | rs28366328 | 1.00E-200 | G | A | 0.9669 | 0.023 | 1707 |
|  | rs1811359 | 3.35E-83 | C | G | -0.2811 | 0.015 | 376 |
|  | rs72928038 | 6.28E-09 | A | G | 0.1068 | 0.018 | 33.7 |
|  | rs3778754 | 4.01E-17 | G | C | 0.1093 | 0.013 | 70.7 |
|  | rs940825 | 3.39E-08 | G | T | 0.1085 | 0.02 | 30.6 |
|  | rs678347 | 3.25E-09 | A | G | -0.0835 | 0.014 | 35.1 |
|  | rs2409780 | 2.23E-11 | C | T | 0.1076 | 0.016 | 44.7 |
|  | rs16903108 | 1.96E-11 | C | T | -0.142 | 0.021 | 44.9 |
|  | rs998731 | 2.00E-08 | T | C | 0.0771 | 0.014 | 31.7 |
|  | rs10985070 | 1.92E-09 | A | C | -0.0786 | 0.013 | 36 |
|  | rs10972201 | 4.28E-13 | A | G | 0.0994 | 0.014 | 52.6 |

**Abbreviations:** SNP: single-nucleotide Polymorphism; SE: standard error; SLE: systemic lupus erythematosus; IBD: inflammatory bowel disease; RA: rheumatoid arthritis.

**Table S3. The results of the heterogeneity test**

| **exposure** | **outcome** | **method** | **Q** | **Q_df** | **Q_pval** |
| --- | --- | --- | --- | --- | --- |
| SLE | Vitiligo | Inverse variance weighted | 96.89643 | 36 | 1.73E-07 |
| Graves' disease | Vitiligo | Inverse variance weighted | 32.62012 | 20 | 0.037122 |
| IBD | Vitiligo | Inverse variance weighted | 296.5571 | 125 | 5.66E-16 |
| Alopecia areata | Vitiligo | Inverse variance weighted | 27.23193 | 9 | 0.00128 |
| Type Ⅰ diabetes | Vitiligo | Inverse variance weighted | 78.99641 | 20 | 5.81E-09 |
| RA | Vitiligo | Inverse variance weighted | 135.5053 | 48 | 2.82E-10 |
| Vitiligo | SLE | Inverse variance weighted | 137.8811 | 29 | 4.06E-16 |
| Vitiligo | Graves' disease | Inverse variance weighted | 77.265 | 35 | 5.05E-05 |
| Vitiligo | IBD | Inverse variance weighted | 58.55011 | 9 | 2.55E-09 |
| Vitiligo | Alopecia areata | Inverse variance weighted | 47.44061 | 33 | 0.049595 |
| Vitiligo | Type Ⅰ diabetes | Inverse variance weighted | 79.37336 | 25 | 1.43E-07 |
| Vitiligo | RA | Inverse variance weighted | 110.3785 | 27 | 4.79E-12 |

**Abbreviations:** SE: standard error; SLE: systemic lupus erythematosus; IBD: inflammatory bowel disease; RA: rheumatoid arthritis.

**Table S4. The results of MR-egger intercept test**

| **exposure** | **outcome** | **Egger-intercept** | **SE** | **P_value** |
| --- | --- | --- | --- | --- |
| SLE | Vitiligo | 0.007385556 | 0.023186301 | 0.751975 |
| Graves' disease | Vitiligo | 0.049957264 | 0.024877693 | 0.059063 |
| IBD | Vitiligo | -0.009543838 | 0.01096569 | 0.385798 |
| Alopecia areata | Vitiligo | -0.030017805 | 0.050652815 | 0.569804 |
| Type Ⅰ diabetes | Vitiligo | 0.074466062 | 0.040558872 | 0.082058 |
| RA | Vitiligo | -0.011811341 | 0.016784571 | 0.48509 |
| Vitiligo | SLE | 0.023567672 | 0.044318889 | 0.599076 |
| Vitiligo | Graves' disease | 0.180740743 | 0.077788307 | 0.026267 |
| Vitiligo | IBD | -0.035839077 | 0.057643099 | 0.551415 |
| Vitiligo | Alopecia areata | 0.014246549 | 0.038530511 | 0.714007 |
| Vitiligo | Type Ⅰ diabetes | -0.005332111 | 0.032328911 | 0.870378 |
| Vitiligo | RA | 0.006422293 | 0.022478727 | 0.777458 |

**Abbreviations:** SE: standard error; SLE: systemic lupus erythematosus; IBD: inflammatory bowel disease; RA: rheumatoid arthritis.

**Table S5. The results of MR PRESSO analyses**

| **exposure** | **outcome** | **MR.Analysis** | **beta** | **se** | **T** | **pval** | **OR** | **or_lci95** | **or_uci95** |
| --- | --- | --- | --- | --- | --- | --- | --- | --- | --- |
| **SLE** | **Vitiligo** | **Raw** | 0.019513 | 0.02914 | 0.669629 | 0.507252 | 1.019705 | 0.963096 | 1.07964 |
|  |  | **Outlier-corrected** | 0.004399 | 0.023157 | 0.189973 | 0.85046 | 1.004409 | 0.95984 | 1.051047 |
| **Graves disease** | **Vitiligo** | **Raw** | -0.00333 | 0.015801 | -0.211 | 0.835026 | 0.996672 | 0.966277 | 1.028022 |
|  |  | **Outlier-corrected** | NA | NA | NA | NA | NA | NA | NA |
| **IBD** | **Vitiligo** | **Raw** | -0.02458 | 0.041534 | -0.59192 | 0.554975 | 0.975715 | 0.899433 | 1.058467 |
|  |  | **Outlier-corrected** | -0.01197 | 0.037461 | -0.31942 | 0.749962 | 0.988105 | 0.918154 | 1.063386 |
| **Alopecia areata** | **Vitiligo** | **Raw** | 0.021689 | 0.052109 | 0.416229 | 0.685247 | 1.021926 | 0.922706 | 1.131817 |
|  |  | **Outlier-corrected** | -0.00203 | 0.038666 | -0.05245 | 0.959203 | 0.997974 | 0.925137 | 1.076546 |
| **TypeⅠdiabetes** | **Vitiligo** | **Raw** | 0.006178 | 0.046902 | 0.131725 | 0.896178 | 1.006197 | 0.917823 | 1.103081 |
|  |  | **Outlier-corrected** | 0.039481 | 0.03091 | 1.277285 | 0.21372 | 1.04027 | 0.979119 | 1.105242 |
| **RA** | **Vitiligo** | **Raw** | 0.153515 | 0.053681 | 2.859753 | 0.005883 | 1.165926 | 1.049485 | 1.295285 |
|  |  | **Outlier-corrected** | 0.115625 | 0.049209 | 2.349687 | 0.02234 | 1.122575 | 1.019361 | 1.236239 |
| **Vitiligo** | **SLE** | **Raw** | 0.019513 | 0.02914 | 0.669629 | 0.507252 | 1.019705 | 0.963096 | 1.07964 |
|  |  | **Outlier-corrected** | 0.004399 | 0.023157 | 0.189973 | 0.85046 | 1.004409 | 0.95984 | 1.051047 |
| **Vitiligo** | **Graves disease** | **Raw** | 0.061543 | 0.094507 | 0.651195 | 0.518644 | 1.063476 | 0.883652 | 1.279894 |
|  |  | **Outlier-corrected** | 0.164088 | 0.090966 | 1.803833 | 0.078986 | 1.178318 | 0.985895 | 1.408297 |
| **Vitiligo** | **IBD** | **Raw** | -0.04781 | 0.036592 | -1.30649 | 0.223783 | 0.953317 | 0.887339 | 1.024201 |
|  |  | **Outlier-corrected** | -0.03335 | 0.030555 | -1.09161 | 0.316889 | 0.967196 | 0.910972 | 1.026889 |
| **Vitiligo** | **Alopecia areata** | **Raw** | 0.125339 | 0.04615 | 2.715889 | 0.009988 | 1.133533 | 1.0355 | 1.240847 |
|  |  | **Outlier-corrected** | NA | NA | NA | NA | NA | NA | NA |
| **Vitiligo** | **TypeⅠdiabetes** | **Raw** | 0.135009 | 0.035503 | 3.802746 | 0.000711 | 1.144547 | 1.067611 | 1.227028 |
|  |  | **Outlier-corrected** | 0.119946 | 0.035625 | 3.366876 | 0.002296 | 1.127436 | 1.051398 | 1.208973 |
| **Vitiligo** | **RA** | **Raw** | 0.079609 | 0.02408 | 3.306029 | 0.002289 | 1.082863 | 1.032943 | 1.135196 |
|  |  | **Outlier-corrected** | 0.082298 | 0.019078 | 4.31386 | 0.000192 | 1.085779 | 1.045929 | 1.127147 |

**Abbreviations:** SE: standard error; SNP: single-nucleotide Polymorphism; OR: odds ratio; SLE: systemic lupus erythematosus; IBD: inflammatory bowel disease; RA: rheumatoid arthritis.

**Table S6. 6 shared risk genes identified in SMR analyses**

| **Gene** | **Disease** | **eQTL data** | **Probe_bp** | **topSNP** | **topSNP_chr** | **Effect allele** | **Other allele** | **Freq** | **b_SMR** | **se_SMR** | **p_SMR** | **p_HEIDI** | **nsnp_HEIDI** | **FDR** |
| --- | --- | --- | --- | --- | --- | --- | --- | --- | --- | --- | --- | --- | --- | --- |
| FCRL1 | Rheumatoid arthritis | eQTLGen | 157777044 | rs7522061 | 1 | C | T | 0.512363 | -0.6392 | 0.106397 | 1.88E-09 | 0.103089 | 20 | 3.46E-07 |
| FCRL1 | Vitiligo | GTEx V8(Whole Blood) | 157764193 | rs10908597 | 1 | C | T | 0.440358 | -0.40999 | 0.117123 | 0.000464 | 0.058295 | 20 | 0.036134 |
| FCRL3 | Rheumatoid arthritis | eQTLGen | 157658459 | rs3761959 | 1 | T | C | 0.5 | 0.109589 | 0.016933 | 9.67E-11 | 0.314974 | 20 | 2.16E-08 |
| FCRL3 | Vitiligo | GTEx V8(Whole Blood) | 157644111 | rs3761959 | 1 | T | C | 0.474155 | 0.431519 | 0.103229 | 2.91E-05 | 0.404284 | 20 | 0.004132 |
| FCRL3 | Vitiligo | eQTLGen | 157658459 | rs2210913 | 1 | T | C | 0.474155 | 0.180626 | 0.041393 | 1.28E-05 | 0.656442 | 20 | 0.002137 |
| FCRL3 | Vitiligo | CAGE | 157648482 | rs3761959 | 1 | T | C | 0.474155 | 0.197378 | 0.045962 | 1.75E-05 | 0.431463 | 20 | 0.00312 |
| FCRL3 | Vitiligo | Westra | 157646476 | rs945635 | 1 | G | C | 0.474155 | 0.209585 | 0.048346 | 1.46E-05 | 0.642826 | 20 | 0.002848 |
| FADS1 | Rheumatoid arthritis | eQTLGen | 61581944 | rs61896141 | 11 | C | A | 0.078297 | -0.14123 | 0.03124 | 6.16E-06 | 0.989938 | 20 | 0.000618 |
| FADS1 | Vitiligo | GTEx V8(Whole Blood) | 61567099 | rs968567 | 11 | T | C | 0.150099 | -0.68456 | 0.18315 | 0.000186 | 0.423166 | 9 | 0.018831 |
| FADS1 | Vitiligo | eQTLGen | 61581944 | rs61896141 | 11 | C | A | 0.152087 | -0.35396 | 0.071667 | 7.85E-07 | 0.368714 | 20 | 0.000158 |
| FADS1 | Vitiligo | CAGE | 61567437 | rs968567 | 11 | T | C | 0.150099 | -0.34426 | 0.073137 | 2.51E-06 | 0.548156 | 20 | 0.000666 |
| FADS1 | Vitiligo | Westra | 61567436 | rs968567 | 11 | T | C | 0.150099 | -0.46965 | 0.098805 | 2.00E-06 | 0.587099 | 20 | 0.000658 |
| FADS2 | Rheumatoid arthritis | eQTLGen | 61597639 | rs968567 | 11 | T | C | 0.079213 | -0.08068 | 0.015138 | 9.83E-08 | 0.999906 | 20 | 1.34E-05 |
| FADS2 | Vitiligo | GTEx V8(Whole Blood) | 61560452 | rs968567 | 11 | T | C | 0.150099 | -0.14782 | 0.030336 | 1.10E-06 | 0.464894 | 20 | 0.00023 |
| FADS2 | Vitiligo | eQTLGen | 61597639 | rs968567 | 11 | T | C | 0.150099 | -0.19858 | 0.040073 | 7.21E-07 | 0.757077 | 20 | 0.000148 |
| FADS2 | Vitiligo | CAGE | 61633933 | rs968567 | 11 | T | C | 0.150099 | -0.43375 | 0.09468 | 4.62E-06 | 0.810773 | 20 | 0.001059 |
| FADS2 | Vitiligo | Westra | 61633932 | rs968567 | 11 | T | C | 0.150099 | -0.63324 | 0.137845 | 4.35E-06 | 0.853627 | 20 | 0.001171 |
| AP003774.1 | Rheumatoid arthritis | eQTLGen | 64094749 | rs479777 | 11 | C | T | 0.250458 | -0.10968 | 0.023243 | 2.37E-06 | 0.427614 | 20 | 0.000258 |
| AP003774.1 | Vitiligo | GTEx V8(Whole Blood) | 64092522 | rs479777 | 11 | C | T | 0.33996 | -0.14993 | 0.035946 | 3.03E-05 | 0.269238 | 20 | 0.00414 |
| AP003774.1 | Vitiligo | eQTLGen | 64094749 | rs479777 | 11 | C | T | 0.33996 | -0.12249 | 0.028796 | 2.10E-05 | 0.588506 | 20 | 0.003254 |
| CCDC88B | Rheumatoid arthritis | eQTLGen | 64116350 | rs510372 | 11 | T | C | 0.280678 | 0.132805 | 0.035043 | 0.000151 | 0.436752 | 20 | 0.010164 |
| CCDC88B | Vitiligo | GTEx V8(Whole Blood) | 64107695 | rs60031276 | 11 | G | A | 0.362823 | 1.36078 | 0.377033 | 0.000307 | 0.144285 | 7 | 0.026696 |
| CCDC88B | Vitiligo | CAGE | 64124544 | rs479552 | 11 | C | G | 0.371769 | 0.188024 | 0.04482 | 2.73E-05 | 0.163029 | 20 | 0.003993 |

**Abbreviations:** eQTL: Expression Quantitative Trait Loci; SE: standard error; SLE: systemic lupus erythematosus; IBD: inflammatory bowel disease; RA: rheumatoid arthritis.

**Table S7. Colocalization analyses**

| **Disease** | **eQTL** | **Chr** | **Start** | **end** | **PP.H0** | **PP.H1** | **PP.H2** | **PP.H3** | **PP.H4** |
| --- | --- | --- | --- | --- | --- | --- | --- | --- | --- |
| Vitiligo | FADS1 | 11 | 61567099 | 61584475 | 0 | 0.000367 | 0 | 0.00837 | 0.991 |
| Vitiligo | FADS2 | 11 | 61583675 | 61634826 | 0 | 0.000367 | 0 | 0.00837 | 0.991 |
| Vitiligo | FCRL3 | 1 | 157646271 | 157670559 | 0 | 0.00572 | 0 | 0.0403 | 0.954 |
| RA | FADS1 | 11 | 61567099 | 61584475 | 0 | 0.000151 | 0 | 0.00769 | 0.992 |
| RA | FADS2 | 11 | 61583675 | 61634826 | 0 | 0.0000999 | 0 | 0.00474 | 0.995 |
| RA | FCRL3 | 1 | 157670559 | 157670559 | 0 | 0.00214 | 0 | 0.0182 | 0.98 |

**Abbreviations:** eQTL: Expression Quantitative Trait Loci; Chr : chromosome; RA: rheumatoid arthritis.

The colocalization analysis evaluates five possible scenarios:

H0: SNPs are not associated with either trait.

H1: SNPs are associated with only one of the traits.

H2: SNPs are associated with only the alternative trait.

H3: SNPs are linked to both traits but do not share the same genetic variants.

H4: SNPs are associated with both traits and share identical genetic variants.

A threshold of PPH4 ≥ 0.8 was set for screening.
